# The Beta Subunit of Non-bifurcating NADH-Dependent [FeFe]-Hydrogenases Differs From Those of Multimeric Electron-Bifurcating [FeFe]-Hydrogenases

**DOI:** 10.3389/fmicb.2020.01109

**Published:** 2020-06-17

**Authors:** Nathaniel A. Losey, Saroj Poudel, Eric S. Boyd, Michael J. McInerney

**Affiliations:** ^1^Department of Plant Biology and Microbiology, The University of Oklahoma, Norman, OK, United States; ^2^Department of Microbiology and Immunology, Montana State University, Bozeman, MT, United States

**Keywords:** [FeFe]-hydrogenase, syntrophy, interspecies electron transfer, NADH-dependent hydrogen production, NADH:quinone oxidoreductase, anaerobe physiology, electron-bifurcating enzymes

## Abstract

A non-bifurcating NADH-dependent, dimeric [FeFe]-hydrogenase (HydAB) from *Syntrophus aciditrophicus* was heterologously produced in *Escherichia coli*, purified and characterized. Purified recombinant HydAB catalyzed NAD^+^ reduction coupled to hydrogen oxidation and produced hydrogen from NADH without the involvement of ferredoxin. Hydrogen partial pressures (2.2–40.2 Pa) produced by the purified recombinant HydAB at NADH to NAD^+^ ratios of 1–5 were similar to the hydrogen partial pressures generated by pure and cocultures of *S. aciditrophicus* (5.9–36.6 Pa). Thus, the hydrogen partial pressures observed in metabolizing cultures and cocultures of *S. aciditrophicus* can be generated by HydAB if *S. aciditrophicus* maintains NADH to NAD^+^ ratios greater than one. The flavin-containing beta subunits from *S. aciditrophicus* HydAB and the non-bifurcating NADH-dependent *S. wolfei* Hyd1ABC share a number of conserved residues with the flavin-containing beta subunits from non-bifurcating NADH-dependent enzymes such as NADH:quinone oxidoreductases and formate dehydrogenases. A number of differences were observed between sequences of these non-bifurcating NADH-dependent enzymes and [FeFe]-hydrogenases and formate dehydrogenases known to catalyze electron bifurcation including differences in the number of [Fe-S] centers and in conserved residues near predicted cofactor binding sites. These differences can be used to distinguish members of these two groups of enzymes and may be relevant to the differences in ferredoxin-dependence and ability to mediate electron-bifurcation. These results show that two phylogenetically distinct syntrophic fatty acid-oxidizing bacteria, *Syntrophomonas wolfei* a member of the phylum *Firmicutes*, and *S. aciditrophicus*, a member of the class *Deltaproteobacteria*, possess functionally similar [FeFe]-hydrogenases that produce hydrogen from NADH during syntrophic fatty acid oxidation without the involvement of reduced ferredoxin. The reliance on a non-bifurcating NADH-dependent [FeFe]-hydrogenases may explain the obligate requirement that many syntrophic metabolizers have for a hydrogen-using partner microorganism when grown on fatty, aromatic and alicyclic acids.

## Introduction

Benzoate and its activated form, benzoyl-CoA, are key intermediates in the microbial degradation of organic matter in anaerobic environments (Harwood et al., 1998; McInerney et al., 2008, 2009). In methanogenic environments, benzoate and other aromatic compounds are degraded to acetate, formate, CO_2_, and H_2_ by syntrophic metabolizers. The microbial conversion of benzoate and other aromatic and fatty acids to acetate, formate, CO_2_, and H_2_ is thermodynamically unfavorable unless formate and H_2_ are kept at low levels. This requires the presence of formate- and H_2_-using microorganisms such as methanogens or sulfate reducers. *Syntrophus aciditrophicus* is a metabolic specialist capable of oxidizing benzoate, alicyclic acids such as cyclohexane-1-carboxylate, and fatty acids when paired with a hydrogen- or formate-consuming partner organism such as a methanogen or sulfate reducer (Jackson et al., 1999; Elshahed and McInerney, 2001). *S. aciditrophicus* also has electrically conductive pili that allow syntrophic growth by direct electron transfer (Walker et al., 2020).

*S. aciditrophicus* syntrophically metabolizes benzoate and cyclohexane-1-carboxylate to acetate, CO_2_, H_2_ and formate via a 6-substituted cyclohex-1-ene-1-carboxyl-CoA intermediate using a common core set of enzymes for all its substrates (James et al., 2019). *S. aciditrophicus* uses an unique mechanism for energy generation from acetyl-CoA where an AMP-forming, acetyl-CoA synthetase is used to make acetate, CoA and ATP from acetyl-CoA, AMP and pyrophosphate (James et al., 2016). During the metabolism of benzoate and cyclohexane-1-carboxylate, reduced electron transfer flavoprotein (EtfAB) and NADH are generated, which must be re-oxidized by the production of hydrogen and/or formate (McInerney et al., 2007, 2009) or by direct electron transfer (Walker et al., 2020). Hydrogen and formate production or direct electron transfer to methanogens from reduced EtfAB requires energy input from a membrane gradient, a process termed reverse electron transfer (McInerney et al., 2007, 2009). A membrane-associated iron-sulfur oxidoreductase in conjunction with a reverse quinone loop and membrane-associated formate dehydrogenase may be responsible for EtfAB re-oxidation in *S. aciditrophicus* similar to the mechanism proposed for *Syntrophomonas wolfei* (Schmidt et al., 2013; Sieber et al., 2015; Crable et al., 2016).

In *S. aciditrophicus*, the re-oxidation of NADH was previously proposed to involve the use of the Rnf complex, which couples the endergonic oxidation of NADH and reduction of ferredoxin with depletion of ionic gradients (McInerney et al., 2007). The reduced ferredoxin would then be used to produce hydrogen or formate (Sieber et al., 2012) by electron bifurcation whereby for every molecule of NADH oxidized, one molecule of reduced ferredoxin is also oxidized (Schut and Adams, 2009). The favorable production of hydrogen or formate from low potential electrons derived from reduced ferredoxin allows the unfavorable production of hydrogen or formate from the high potential electrons from NADH (Schut and Adams, 2009). If *S. aciditrophicus* utilizes electron-bifurcating (BF) [FeFe]-hydrogenases and formate dehydrogenases, *S. aciditrophicus* would have to use the Rnf complex to make reduced ferredoxin from electrons derived from NADH oxidation, which would consume much energy, leaving less energy for growth (Seedorf et al., 2008; Biegel and Müller, 2010). An alternative possibility would be the production of hydrogen from NADH without electron bifurcation or involvement of ferredoxin, as proposed for Hyd1ABC from *Syntrophomonas wolfei* (Losey et al., 2017). A non-bifurcating (non-BF) NADH-dependent enzyme could only produce hydrogen at much lower partial pressures (<60 Pa) than that of BF [FeFe]-hydrogenases, however, with a suitable hydrogen-consuming partner such as a hydrogenotrophic methanogen maintaining a hydrogen partial pressure below 60 Pa, the process would be thermodynamically favorable.

The genome of *S. aciditrophicus* encodes two predicted hydrogenases, a cytoplasmic [NiFe]-hydrogenase and a dimeric cytoplasmic [FeFe]-hydrogenase (HydAB) (McInerney et al., 2007). The latter is predicted to use NADH as the electron donor. The genes for both hydrogenases were expressed when *S. aciditrophicus* was grown in pure culture on crotonate and in coculture with methanogen on crotonate, benzoate, and cyclohexane-1-carboxylate (Sieber et al., 2014) but only HydAB peptides were detected in the proteome under these growth conditions (James et al., 2019).

Here, we determine if HydAB produces hydrogen from NADH and whether this process requires reduced ferredoxin. We then compare BF and non-BF NADH-dependent hydrogenases to identify differences that can be used to distinguish members of these two groups of enzymes that may be relevant to the differences in ferredoxin-dependence and the ability to mediate electron-bifurcation. To produce an active form of HydAB from *S. aciditrophicus*, a recombinant DNA approach was used to produce HydAB in *Escherichia coli* which was then purified and characterized.

## Materials and Methods

### Plasmid Design

A DNA sequence encoding the genes for the *S. aciditrophicus* dimeric [FeFe]-hydrogenase HydAB (Locus Tags: SYN_01369 and SYN_01370, Genome Genbank ID: CP000252) was ordered from Integrated DNA Technologies (Coralville, IA, United States) and delivered in a plasmid for recombinant protein expression, including a T7 promoter region for protein expression. This plasmid was designated as pIDTKan-SbHydAB. An additional amino acid sequence including six histidine residues (MGSSHHHHHHSQDPNSSSARL, predicted 2.4 kDa molecular mass) was added to the N-terminal region of SYN_01370 to allow for nickel affinity purification of the protein product. A homology search of the *Syntrophus aciditrophicus* genome using the *Clostridium pasteurianum* ferredoxin (GenBank Nucleotide ID: M11214) as the search query identified SYN_03059 as the most similar match. A DNA sequence encoding for SYN_03059 was also ordered from Integrated DNA Technologies for recombinant protein expression and the plasmid referred to as pIDTAmp-SbFd.

### Expression and Purification of Recombinant Proteins

*E. coli* BL21(DE3) cells were co-transformed with pIDTKan-SbHydAB and pCDFDuet-1 SwHydEFG (Losey et al., 2017) and used for expression. The latter plasmid contains maturation genes needed for the expression of a functional HydAB protein. The cells were grown at pH 7.5 in LB medium containing 50 mM potassium phosphate, 10 g ⋅ l^–1^ glucose, and the appropriate selective antibiotics. Expression conditions were similar to those used previously for the expression of the trimeric *S. wolfei* hydrogenase but with different iron and cysteine concentrations (Losey et al., 2017). Briefly, cultures were incubated aerobically to an OD of 0.4–0.6 followed by induction with the addition of 0.5 mM isopropyl β-d-1-thiogalactopyranoside (IPTG). After induction, 10 mM sodium fumarate, 0.5 mM cysteine, and 0.5 mM ferric ammonium citrate were added. Cultures were then purged with nitrogen overnight to generate anaerobic conditions and harvested by centrifugation (6,000 ⋅ *g*; 20 min; 4°C). Cell pellets were stored in liquid nitrogen until used. After induction, all additional manipulations of cell pellets and protein fractions were performed under anaerobic conditions including the use of a Coy anaerobic chamber operated with a 95–99% nitrogen and 1–5% hydrogen atmosphere. Protein-containing fractions were maintained under anaerobic conditions at all times. Cell breakage and centrifugation steps were performed outside of the anaerobic chamber, but the fractions were loaded inside of the anaerobic chamber and kept anaerobic by using centrifuge bottles sealed with caps containing o-rings and collecting cell extracts into a sealed tube with an anaerobic atmosphere connected to the pressure cell via tubing and a needle. Protein chromatography and fraction collection were performed inside of an anaerobic chamber.

BL21(DE3) cells (3 g) induced for HydAB expression were resuspended in lysis buffer (50 mM potassium phosphate (pH 7.5), 0.5 M NaCl, 2 mM dithioerythritol (DTE), 5 μM FAD, 5 μM FMN, 20 mM imidazole, 0.5 mg lysozyme, and 0.1% Triton X-100). The cells were passed through a French pressure cell operated at 140 MPa (Megapascals) and the resulting fluid was clarified by centrifugation (13,000 ⋅ g; 10 min; 4°C). The cell-free extract was then loaded onto a HisTrap Hp 5 ml column (GE Healthcare Life Sciences, Pittsburgh, PA, United States) with an imidazole concentration of 20 mM, washed with 50 mM imidazole, and eluted with 250 mM imidazole. The 250 mM imidazole fraction was concentrated using an Amicon Ultra 0.5 ml centrifugal filter with a 10 kDa molecular weight cut-off (MWCO) filter, designated as the HydAB Fraction and stored in liquid nitrogen until used for further analyses.

*E. coli* BL21(DE3) cells (4.5 g) transformed with pIDTAmp-SbFd were grown and induced using the same conditions as those used for production of *S. aciditrophicus* HydAB. The cells were lysed using a French pressure cell and the recombinant SYN_01370 gene product was purified using nickel affinity chromatography as described for HydAB.

### Partial Purification of Ferredoxin From *S. aciditrophicus*

Approximately 5 g of wet cell mass of *S. aciditrophicus* were suspended in lysis buffer and disrupted by passage through a French pressure cell operating at 140 MPa. The lysis buffer consisted of 25 mM potassium phosphate (pH 6.5) with 2 mM DTE, 5 mg ⋅ l^–1^ lysozyme, 0.125 mg ⋅ l^–1^ DNase and 0.5% Triton X-100. The resulting extract was clarified by centrifugation (12,000 *g*; 10 min; 4°C) and loaded onto a DEAE-Sepharose anion exchange column and eluted with a gradient from 0 to 1.0 M NaCl. A dark brown fraction that was last to elute from the column was collected and passed through a 100 kDa MWCO filter to remove high molecular mass proteins. The filtrate was then concentrated and desalted using a 10 kDa MWCO filter.

### Enzymatic Assays

Enzyme activity measurements were determined in triplicate with various concentrations of protein to ensure activity was proportional to the amount of protein added unless otherwise noted. Hydrogen-oxidizing assays were performed in 1.4 ml quartz cuvettes (Nova Biotech, El Cajon, CA, United States) with gas tight rubber stoppers. The assays were performed in 1 ml volumes at 37°C using an assay buffer (50 mM Tris pH 7.5, 2 mM DTE, 5 μM FMN, and 5 μM FAD). NAD(P)^+^ reduction was tested using 1 mM NAD^+^ or 1 mM NADP^+^ with and without 10 μM clostridial ferredoxin. Hydrogen-dependent clostridial ferredoxin reduction was tested using 30 μM clostridial ferredoxin. Cuvettes that were never exposed to viologen dyes were used for all assays except those involving methyl viologen, which were conducted with a separate set of cuvettes. Hydrogen-dependent reduction of methyl viologen was monitored using 10 mM methyl viologen as electron acceptor. Hydrogen-oxidizing assays were initiated with the addition of hydrogen at a pressure of 1.2 × 10^5^ Pa to a 100% nitrogen headspace. NAD^+^ and NADP^+^ reduction was followed at 340 nm (ε_340_ = 6.2 mM^–1^ cm^–1^); ferredoxin reduction was followed at 430 nm (ε_430_ = 13.1 mM^–1^ cm^–1^); and methyl viologen reduction was followed at 600 nm (ε_600_ = 10.0 mM^–1^ cm^–1^).

Hydrogen production assays were conducted using 6.5 ml serum bottles with a 1.0 ml liquid assay volume with shaking (200 rpm) at 37°C. The reaction buffer contained 50 mM Tris (pH 7.5), 2 mM DTE, 5 μM FMN, and 5 μM FAD. Hydrogen production was tested with 1 mM NADH alone or with a reduced ferredoxin-generating system consisting of 0.1 U pyruvate:ferredoxin oxidoreductase, 10 mM pyruvate, 0.1 mM thiamine pyrophosphate, 1 mM CoA and either 0.5 μM ferredoxin partially purified from *S. aciditrophicus*, 5.0 μM of recombinantly produced *S. aciditrophicus* ferredoxin, or 20 μM clostridial ferredoxin. Hydrogen production from reduced ferredoxin using the ferredoxin-generating system in the absence of NADH was also tested.

Hydrogen production assays to determine equilibrium hydrogen concentration at different NADH/NAD^+^ ratios were performed using 6.5 ml serum bottles with a 1.0 ml liquid assay volume. Reaction buffer consisted of 10 mM Tris (pH 7.5), 2 mM DTE, 5 μM FMN, and 5 μM FAD. The total concentration of NADH and NAD^+^ was held constant at 6 mM with variable NADH/NAD^+^ ratios of 5.0, 1.0, and 0.2. Hydrogen production assay bottles were incubated for 24 h at room temperature before measurement.

### Growth of Cultures for Hydrogen Partial Pressure Measurements

Hydrogen concentrations were measured from triplicate cultures of either pure cultures of *S. aciditrophicus* or cocultures of *S. aciditrophicus* and *M. hungatei* grown on the following substrates: 20 mM crotonate, 2.5 mM sodium benzoate, or 2.5 mM cyclohex-1-ene-1-carboxylate. The basal medium (Tanner, 2007) consisted of a mineral solution (10 ml ⋅ l^–1^), a trace metal solution (5 ml ⋅ l^–1^), a vitamin solution (10 ml l^–1^), a cysteine-sulfide solution (0.05%), 1 mg ⋅ l^–1^ of resazurin, and 3.5 g ⋅ l^–1^ of NaHCO_3_. The medium was prepared anaerobically under a N_2_/CO_2_ (80:20) atmosphere. Each culture consisted of 20 ml of medium in a 160 ml serum bottle. The inoculum was a 10–15% (v/v) transfer of *S. aciditrophicus* grown in pure culture on 20 mM crotonate in the basal medium (culture O.D. = 0.54). Cocultures were created by addition of 2.0–2.5% (volume to volume) of a *M. hungatei* culture grown on H_2_/CO_2_ (80:20) in the basal medium with 0.05% sodium acetate as described previously (Sieber et al., 2014). Cultures were grown at 37°C but allowed to equilibrate to room temperature before hydrogen measurements were taken. Crotonate, benzoate and cyclohex-1-ene-1-carboxylate concentrations were determined every 6 days while hydrogen and methane concentrations were determined every 3 days.

### Analytical Techniques

Sodium dodecyl sulfate polyacrylamide gel electrophoresis (SDS-PAGE) and non-denaturing polyacrylamide gel electrophoresis (Native PAGE) analyses were performed using precast 8–16% Tris-Glycine gels (Life Technologies Co., Carlsbad, CA, United States) according to manufacturer’s instruction and stained using Coomassie Brilliant Blue G-250. The Bradford protein assay (Life Technologies Co., Carlsbad, CA, United States) was used to determine protein concentrations with bovine serum album as the standard. Peptide identification was performed at the Laboratory for Molecular Biology and Cytometry Research at OUHSC (Oklahoma City, OK, United States). Peptides were digested by trypsin followed by high-performance, liquid chromatography-tandem mass spectrometry (HPLC-MS/MS). Peptides were identified by Mascot search of the NCBI non-redundant (nr) protein database.

The iron content, flavin content, and molecular mass of HydAB was determined as described previously for Hyd1AB from *S. wolfei* (Losey et al., 2017). Briefly, iron content determination was performed using the ferrozine assay (Riemer et al., 2004), flavin content was determined by HPLC analysis using a UV detector (Seedorf et al., 2004), and the molecular mass was determined by size-exclusion chromatography using a Superdex 200 10/300 GL (GE Healthcare Life Sciences) calibrated with gel filtration standards (Bio-Rad Laboratories, Hercules, CA, United States).

Crotonate, benzoate and cyclohex-1-ene-1-carboxylate concentrations were determined by high performance liquid chromatography (HPLC) analysis and methane was measured using a gas chromatograph with a flame ionization detector (Sieber et al., 2014). Hydrogen concentrations were determined by comparison against hydrogen standards (0.01–1.0%) using a gas detector (Peak Performer RCP-910, Peak Laboratories, Mountain View, CA, United States) as described previously (Seiler et al., 1980).

### Reagents and Chemicals

NADH, NAD^+^, FMN, FAD, pyruvate, thiamine pyrophosphate, methyl viologen, and coenzyme A were purchased from Sigma-Aldrich (St. Louis, MO, United States).

### Sequence Comparisons

High sequence homology between the *T. maritima* BF [FeFe]-hydrogenase beta subunit and the *E. coli* NuoF was previously reported (Verhagen et al., 1999). The *S. aciditrophicus* HydB sequence shares homology with the *T. maritima* [FeFe]-hydrogenase BF beta subunit (39.7% sequence identity), *E. coli* NuoF (44.1% sequence identity), and *R. capsulatus* formate dehydrogenase (FDH) beta subunit (39.4% sequence identity). To identify sequence differences that may relate to the capacity for BF, beta subunits (NuoF/Nqo1 homologs) from nine non-BF NADH-dependent enzymes (five FDH, two [FeFe]-hydrogenases, and two NADH:quinone oxidoreductases), and eight known BF [FeFe]-hydrogenases and FDHs were compared. Five beta subunit sequences of known BF enzymes included the [FeFe]-hydrogenases from *Thermotoga martima* (Schut and Adams, 2009), *Acetobacterium woodii* (Schuchmann and Muller, 2012), *Moorella thermoacetica* (Wang et al., 2013c), *Ruminococcus albus* (Zheng et al., 2014), *Desulfovibrio. fructosovorans* (Kpebe et al., 2018), and *Caldanaerobacter. tengcongensis* (Soboh et al., 2004), the FDH from *Clostridium acidurici* (Wang et al., 2013b) and a [FeFe]-hydrogenase-formate dehydrogenase complex from *Clostridium autoethanogenum* (Wang et al., 2013a). Beta subunit sequences from non-BF NADH-dependent FDHs from *Cupriavidus oxalaticus* (previously *Pseudomonas oxalaticus*) (Ruschig et al., 1976; Muller et al., 1978), *Methylosinus trichosporium* (Jollie and Lipscomb, 1991), *Cupriavidus eutropha* (previously *Alcaligenes eutropha)* (Friedebold and Bowien, 1993), *Methylobacterium extorquens* (Laukel et al., 2003), and *Rhodobacter capsulatus* (Hartmann and Leimkühler, 2013) were included. NADH-dependent:quinone oxidoreductase subunits from *Escherichia coli* NuoF and *T. thermophilus* Nqo1 were also included since the crystal structure of *T. thermophilus* Nqo has been solved and has been used as the basis for homology modeling of a FDH and [FeFe]-Hydrogenase (Sazanov and Hinchliffe, 2006; Hille et al., 2014; Chongdar et al., 2019).

The 17 protein sequences for the beta subunits from aforementioned enzymes were retrieved from Genbank (see Supplementary Tables S1–S3 for accession numbers) and aligned using MEGA X (Kumar et al., 2018) with default Clustal W alignment setting (Thompson et al., 1994). The conserved residues present in beta subunit sequences of BF enzymes were then manually compared to the beta subunit sequences of NADH-dependent enzymes to identify residues that differed between the two groups. Three regions were identified that had different residues in the non-BF NADH-dependent beta subunits sequences compared to the BF beta subunit sequences: one near the NADH binding site, one near the FMN binding site, and one within the soluble-ligand-binding-beta-grasp (SLBB) domain (Burroughs et al., 2007). These three differential regions between the beta subunits of BF and non-BF NADH-dependent enzymes were used to identify additional homologs that were likely to be non-BF NADH-dependent enzymes using a previously compiled database comprising 137 trimeric (group 2) and 72 tetrameric (group 3) [FeFe]-hydrogenase homologs (Poudel et al., 2016). A phylogenetic analysis of all of the compiled beta subunit sequences (227 amino acid sequences; see Supplementary Tables S1–S3) was conducted by first aligning the sequences with COBALT specifying default settings (Papadopoulos and Agarwala, 2007). The alignment block was then manually trimmed to 495 positions from the full alignment of up to 1930 positions (Nqo1 residues 1-425) such that biased phylogenetic signal from non-overlapping/aligned positions was minimized. A maximum likelihood phylogenetic reconstruction was performed using the JTT matrix-based substitution model (Jones et al., 1992), as implemented within MEGA X (Kumar et al., 2018).

## Results

### Purification and Characterization of *S. aciditrophicus* Hydrogenase HydAB

The protein products of SYN_01369 and SYN_01370 were recombinantly produced in *E. coli* and purified using nickel-affinity chromatography and molecular mass cut off filters. Recombinant HydAB was purified 36-fold with a 62% yield and a final specific activity of 13.0 U ⋅ mg^–1^ when assayed with the artificial electron donor methyl viologen (Supplementary Table S4). Other reported specific activities values for methyl viologen reduction with hydrogen from recombinantly produced multimeric [FeFe]-hydrogenases include: 571 U ⋅ mg^–1^ for *S. wolfei* Hyd1ABC (Losey et al., 2017), 2500 U ⋅ mg^–1^ for *D. fructosovorans* HndABCD (Kpebe et al., 2018), and 500 U ⋅ mg^–1^ for *T. maritima* HydABC (Chongdar et al., 2019). The specific activity for methyl viologen reduction with hydrogen for multimeric [FeFe]-hydrogenases purified from native organisms were: 70 U ⋅ mg^–1^ for *T. maritima* HydABC (Schut and Adams, 2009), 760 U ⋅ mg^–1^ for *A. woodii* HydABCD (Schuchmann and Muller, 2012), 181 U ⋅ mg^–1^ for *M. thermoacetica* HydABC (Wang et al., 2013c) and 18000 U ⋅ mg^–1^ for *C. autoethanogenum* FdhA/HytABCDE (Wang et al., 2013a). All further enzymatic assays and properties were determined using this fraction (HydAB fraction).

SDS-PAGE and Native PAGE analyses showed that a few contaminants were present in the final purified fraction. SDS-PAGE analysis (Supplementary Figure S1) showed two main bands, which were estimated to be 68 and 64 kDa in size that closely matched the predicted molecular masses from their respective amino acid sequences of HydA (68.5 kDa with N-terminal His tag) and HydB (66 kDa). Peptide analysis of the HydAB fraction identified peptides matching HydA and HydB along with peptides from *E. coli* proteins (Supplementary Table S5). Molecular mass determination by size exclusion chromatography indicated a protein of 287 kDa, which is consistent with a dimer of the αβ heterodimer of the HydAB subunits, i. e., a α_2_β_2_ heterotetramer. Native PAGE (Supplementary Figure S2) detected a band migrating at a slightly larger molecular mass of 322 kDa. The iron content of the purified recombinant HydAB was 71.7 ± 15.4 moles of iron per mole per heterotetramer (287 kDa) or 35.9 ± 7.7 moles of iron per mole of the single 144 kDa αβ heterodimer. Based on domain analysis, HydAB is predicted to contain five [4Fe-4S]-clusters, two [2Fe-2S]-clusters, and six Fe in the [H-Cluster] for an iron content of 30 moles of iron per mole of αβ heterodimer, which is slightly lower than the experimentally determined iron content of 35.9 ± 7.7 moles of iron per mole of the 143.5 αβ heterodimer. The flavin content per dimer of the αβ heterodimer was 1.06 mole of flavin mononucleotide (FMN) per mole of a 287 kDa complex or 0.5 FMN per 144 kDa αβ heterodimer.

### Purification and Characterization of *S. aciditrophicus* Ferredoxin

A protein fraction that contained *S. aciditrophicus* ferredoxin eluted at 0.62 M NaCl from a DEAE Sepharose column. The ferredoxin-containing fraction was dark brown and had a 390/280 absorbance ratio of 0.082, which is much lower than the 390/280 absorbance ratio of 0.7–0.8 reported for clostridial ferredoxin (Schonheit et al., 1978). Using the extinction coefficient of characterized clostridial ferredoxins (30,000 cm^–1^ M^–1^ at 390 nm) (Hong and Rabinowitz, 1970; Schonheit et al., 1978) the concentration of the *S. aciditrophicus* ferredoxin fraction was 3.7 μM with a total protein concentration of 0.25 mg ⋅ ml^–1^. Peptide analysis of this fraction showed a large number of matches and high sequence coverage to the SYN_03059 gene product (Supplementary Table S6). SYN_03059 annotates as a ferredoxin with two [4Fe-4S] clusters, a predicted molecular mass of 6.01 kDa, and a pI of 3.63.

In a separate purification, the SYN_03059 gene product recombinantly produced in *E. coli* was eluted during nickel affinity chromatography at an imidazole concentration of 250 mM and had a dark brown color. The 390/280 absorbance ratio of the nickel affinity-purified fraction was 0.63 with an estimated ferredoxin concentration of 34 μM. SDS-PAGE analysis of the ferredoxin partially purified from *S. aciditrophicus* and ferredoxin recombinantly produced in *E. coli* had a molecular mass of 12 kDa, roughly twice that predicted from the amino acid sequence (Supplementary Figure S3). This difference suggests that the ferredoxin is a homodimer or there was anomalous migration as a result of interactions of the negatively charged peptides and SDS micelles (Darimont and Sterner, 1994; Huang et al., 2016; Kpebe et al., 2018).

### Enzyme Activities of the Purified Recombinant *S. aciditrophicus* HydAB

**FIGURE 1:**
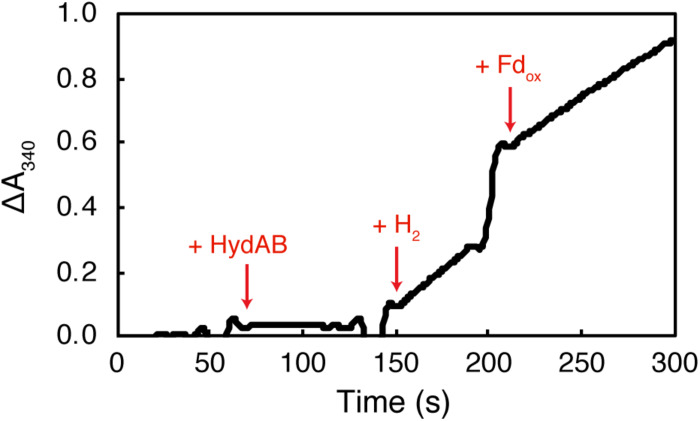
The reduction of NAD^+^ by HydAB is not ferredoxin dependent. Purified recombinant HydAB (8.1 μg) was added to assays as denoted by + HydAB. The reaction was initiated with the addition of H_2_. Clostridial ferredoxin was added at a concentration of 10 μM as indicated by + Fd_ox_. The rate of reduction of NAD^+^ (ΔA340) after the addition of H_2_ (0.275 ΔA ⋅ min^–1^) was similar to the change after the addition of clostridial ferredoxin (0.23 ΔA ⋅ min^–1^).

The purified recombinant HydAB reduced methyl viologen (specific activity of 13.0 Umg^–1^) and NAD^+^ (specific activity of 4.75 Umg^–1^) with hydrogen as the electron donor (Table 1). Characterized BF hydrogenases require the addition of oxidized ferredoxin before significant reduction of NAD^+^ occurs (Schuchmann and Muller, 2012; Wang et al., 2013a). In contrast, the rate of NAD^+^ reduction by purified recombinant *S. aciditrophicus* HydAB was not affected by the presence of oxidized ferredoxin (Figure 1). The specific NAD^+^ reduction activity of HydAB was 4.8 U ⋅ mg^–1^ of protein in the absence of the clostridial ferredoxin and 3.8 U ⋅ mg^–1^ of protein in its presence. Similarly, the specific NAD^+^ reduction activity of HydAB was 1.6 U ⋅ mg^–1^ in the absence of the purified recombinant *S. aciditrophicus* ferredoxin and 1.7 U ⋅ mg^–1^ in its presence. The reduction of NADP^+^ by purified recombinant HydAB was not observed either in presence or the absence of clostridial ferredoxin. In addition, neither clostridial ferredoxin nor purified recombinant *S. aciditrophicus* ferredoxin was reduced by HydAB with hydrogen in either the presence or absence of NAD^+^ (Table 1). The dependence of NAD^+^ reduction on the presence of the purified recombinant *S. aciditrophicus* ferredoxin was tested only in a single assay due to the limited supply of this protein.

**TABLE 1:** Specific activities of purified recombinant HydAB.

Reaction	Hydrogenase activity (U/mg)^a^
H_2_ → MV_ox_	13.0
H_2_ → NAD^+^	4.8
H_2_ → NAD^+^ + Clostridial Fd_ox_^c^	3.8^b^
H_2_ → NADP^+^	<0.01
H_2_ → NADP^+^ + Clostridial Fd_ox_^c^	<0.01
H_2_ → Clostridial Fd_ox_^c^	<0.01
MV_red_ → H_2_	0.42
NADH → H_2_	0.027
NADH + Clostridial Fd_red_^c^ → H_2_	0.014
NADH + *Syntrophus* Fd_red_^c^ → H_2_	0.018
Clostridial Fd_red_^c^ → H_2_	<0.001

*^*a*^Assays were performed at 37°C and a pH of 7.5; 1 unit of activity (U) equals 2 μmol of electrons transferred per min. ^*b*^The activity was measured as the rate of NAD^+^ reduction (Δ 340 nm) in the presence of Fd_*ox*_, and not the rate of Fd_*ox*_ reduction (Δ 430 nm), which was not observed to occur. ^*c*^*Syntrophus* Fd_*red*_ refers to recombinantly produced S. aciditrophicus ferredoxin. Clostridial Fd_*red*_ refers to ferredoxin purified from *C. pasteurianum*.*

**FIGURE 2:**
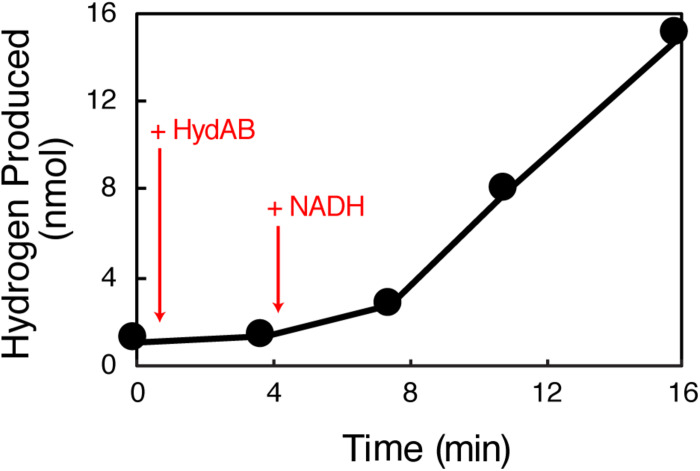
Production of hydrogen from NADH by HydAB independent of reduced ferredoxin. The enzyme assay was performed with 50.7 μg HydAB (+HydAB). NADH was added to a final concentration of 1 mM (+NADH). The maximal rate of hydrogen production in the enzyme assay was 30.1 nanomoles ⋅ min^–1^ ⋅ mg^–1^.

HydAB produced hydrogen from methyl viologen reduced with dithionite (specific activity of 0.42 U ⋅ mg^–1^) and from NADH (specific activity of 0.03 U ⋅ mg^–1^) (Table 1). The latter assays used separate cuvettes not previously exposed to methyl viologen. In contrast to known BF hydrogenases (Schut and Adams, 2009; Schuchmann and Muller, 2012; Wang et al., 2013c), the specific rate of hydrogen production from NADH was not enhanced by the presence of a reduced ferredoxin-generating system consisting of clostridial pyruvate:ferredoxin oxidoreductase, pyruvate, and clostridial ferredoxin (Table 1). The maximum observed hydrogen production rate in the absence of the reduced ferredoxin-generating system was 1.5 nmol min^–1^ (Figure 2). This compares to a rate of 0.6 nmol min^–1^ rate in the presence of both a reduced ferredoxin generating system and NADH (Supplementary Figure S4). Neither the use of purified recombinant *S. aciditrophicus* ferredoxin (specific activity of 0.018 U ⋅ mg^–*I*^) nor ferredoxin partially purified from *S. aciditrophicus* cells enhanced hydrogen production activity (Table 1). Due to the limited yield of ferredoxin partially purified from *S. aciditrophicus*, only a single hydrogen production assay with a specific activity of 0.013 U ⋅ mg^–1^ was performed. The results showed that regardless of the source of ferredoxin source, no enhancement of hydrogen production rate was observed.

Equilibrium hydrogen partial pressures produced by HydAB varied with the NADH/NAD^+^ ratio (Table 2), ranging from 40.2 (± 3.2) Pa at the highest tested NADH/NAD^+^ ratio of 5.0–2.2 (± 0.4) Pa at a NADH/NAD^+^ ratio of 0.2. These values are much lower than the equilibrium hydrogen partial pressures of 1,000 Pa produced by BF hydrogenases (equivalent to E′ = −367 mV). The hydrogen partial pressure generated by HydAB never reached the thermodynamically predicted equilibrium pressure for the given NADH/NAD^+^ ratio at pH 7.5 (Table 2). A small amount of hydrogen was observed in the absence of NADH addition, which was likely due to carryover from manipulation of the enzyme inside the anaerobic chamber that contained hydrogen in the atmosphere.

**TABLE 2:** Equilibrium hydrogen concentrations produced by HydAB with varied NADH/NAD^+^ ratios.

Condition^a^	Ratio of NADH/NAD^+^	Predicted equilibrium hydrogen partial pressure (Pa)^d^	Observed hydrogen partial pressure (Pa)^b^	Hydrogen produced (nanomol)^b^
1.0 mM NAD^+^ + 5.0 mM NADH	5.0	105.9	40.2 ± 3.2	101.8 ± 8.1
3.0 mM NAD^+^ + 3.0 mM NADH	1.0	21.3	9.1 ± 2.4	23.2 ± 6.0
5.0 mM NAD^+^ + 1.0 mM NADH	0.2	4.24	2.2 ± 0.4	5.4 ± 1.1
0.0 mM NAD^+^ + 0.0 mM NADH	NA^c^	NA	0.4 ± 0.2	1.0 ± 0.6

*^*a*^Assays were performed at pH 7.5 in 6.5 ml serum bottles with 1.0 ml of liquid volume containing 69.6 μg of HydAB and 5.5 ml of headspace. ^*b*^Values are the mean ± the standard deviation of triplicate assays. ^*c*^NA, not applicable. ^*d*^Predicted equilibrium concentrations of hydrogen were calculated as described by Gutekunst and Schulz (2018). Equation and terms used were: e^((–0.094⋅n ⋅F)/(R⋅T))^ [NADH/NAD^+^] ⋅ 10^(–pH +7)^ = [pH_2_]. *F* (Faraday’s constant) = 96.485 J ⋅ V^–1^ ⋅ mol^–1^ of electron equivalents. *R* (Gas Constant) = 8.314 J ⋅ K ⋅ mol^–1^. *n* = 2, number of e^–^ in transfer reaction. *T* = 298.15 Kelvin. [pH_2_] = Partial pressure of hydrogen −0.094 = ΔE′, difference in standard reduction potentials of [H^+^/H_2_] = −414 mV and [NADH/NAD^+^] = −320 mV.*

Hydrogen partial pressures observed during the metabolism of crotonate, cyclohex-1-ene-1- carboxylate, and benzoate by pure cultures of *S. aciditrophicus* ranged from 8.3 to 36.6 Pa while partial pressures reached by cocultures of *S. aciditrophicus* and *M. hungatei* were between 6.6 and 19.1 Pa (Supplementary Figure S5), similar to those generated by HydAB operating at NADH/NAD^+^ ratios of 1.0 or higher (9.1–40.2 Pa) (Table 2).

### Beta Subunits of Non-BF NADH-Dependent [FeFe]-Hydrogenases Differ From Those of BF Enzymes

**FIGURE 3:**
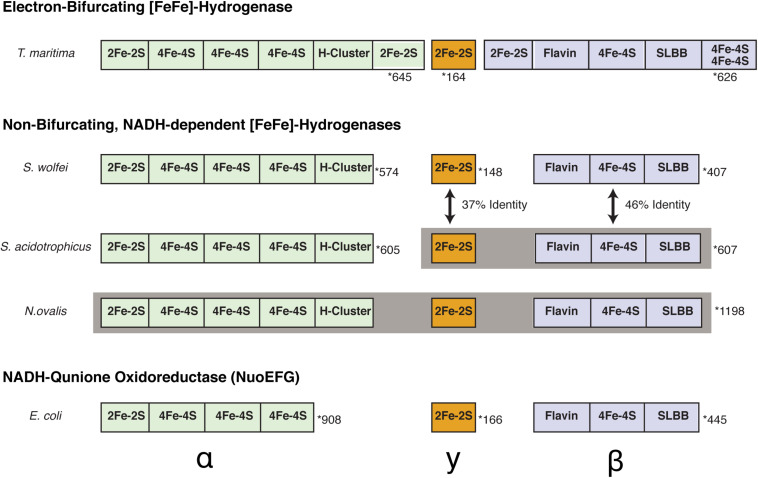
Comparison of subunit compositions and domains between *S. aciditrophicus* HydAB, *S. wolfei* Hyd1ABC, and *N. ovalis* [FeFe]-hydrogenases. Cofactor binding sites are orientated from the N-terminal to C-terminal portion of each subunit. Gray shading indicates subunits that are fused together into a single polypeptide chain. The number of total amino acid residues for each is displayed to the right and is marked with a star. The peptides and domains are not scaled proportionally to accurately reflect number of amino acids in each subunit.

**FIGURE 4:**
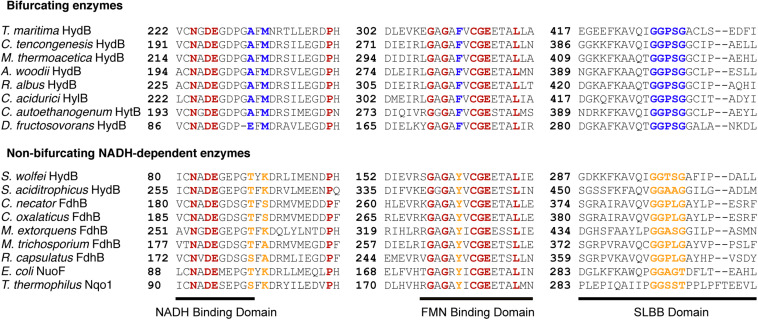
Differences in conserved residues near NADH and FMN binding-sites and the SLBB conserved region of the beta subunits of BF and non-BF NADH-dependent enzymes. The solid black line indicates residues that overlap with proposed NADH and FMN binding sites in *T. thermophilus* Nqo1 (Walker, 1992; Sazanov and Hinchliffe, 2006). Numbers indicate amino acid residue positions in the respective beta subunit amino acid sequences. Red highlights amino acid residues that are conserved in all 17 sequences. Blue highlights residue positions that are conserved in BF beta subunits. Yellow highlights the equivalent residues from alignments of beta subunits from non-BF NADH-dependent enzymes.

**FIGURE 5:**
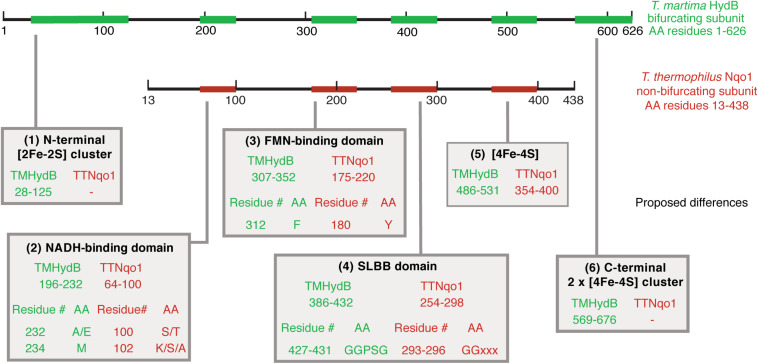
Summary of proposed criteria to differentiate BF enzymes from non-BF NADH-dependent enzymes. An overlay of the *T. maritima* HydB (green) and the *T. thermophilus* Nqo1 (red) sequences is shown. The location of the proposed differential criteria that demarcate beta subunits from BF and non-BF NADH-dependent enzymes are numbered at the bottom from (1) to (6) and the relevant region of the sequence is indicated by arrows.

Next, we then compared the amino acid sequences of BF and non-BF NADH-dependent hydrogenases to identify differences that can be used to distinguish members of these two groups of enzymes and that may be relevant to the ability to mediate electron-bifurcation. We identified several differences that distinguish beta subunits of BF enzymes from the beta subunits of non-BF NADH-dependent enzymes. The first difference is that BF enzymes contain additional [Fe-S] cluster binding domains compared to non-BF NADH-dependent enzymes (Figure 3 and Supplementary Table S7). However, both BF and non-BF NADH-dependent enzymes display substantial variation in the arrangement, type, and number of [Fe-S] cluster binding domains making it difficult to distinguish between the two types of enzymes based solely on the number of [Fe-S] cluster-binding domains in the protein sequence (Figure 3). Yet, the other three differences appear to be more diagnostic of putative BF and non-BF enzymes with one pattern for the BF enzyme beta subunits and another pattern for the non-BF beta subunits (Figures 4, 5). For example, key differences in BF and non-BF beta subunits are observed in a region of the enzyme that, in the case of Nqo1 from *T. thermophilus*, binds NADH (Sazanov and Hinchliffe, 2006; Sazanov, 2007). Specifically, the beta subunits of BF enzymes include an alanine or glutamic acid at position 232 of the *T. maritima* HydB sequence and a methionine at position 234 of this same sequence (Figures 4, 5). In contrast, non-BF enzymes have a threonine or serine residue at the 232 position and lysine, serine, or alanine at the 234 position. The second difference among BF and non-BF enzymes occurs near the FMN binding site. Here, the beta subunits of BF enzymes have phenylalanine at the position equivalent to 367 of *T. maritima* HydB whereas the non-BF enzymes have a tyrosine at this position (Figures 4, 5). Intriguingly, in the case of the Nqo1 from *T. thermophilus*, a tyrosine at this position is believed to facilitate NADH binding through hydrogen bonding (Sazanov and Hinchliffe, 2006; Burroughs et al., 2007). The third major difference occurs near the proposed cofactor-binding SLBB domain (*T. maritima* HydB positions 427–431). All beta subunits of putative BF enzyme have the sequence glycine-glycine-proline-serine-glycine (GGPSG) which is not found in solely NADH-dependent enzymes (Figures 4, 5). The role of the highly conserved residues in the SLBB domain is not known but SLBB domains in other proteins are proposed to bind soluble cofactors (Burroughs et al., 2007). While this domain may play a similar role in BF enzymes, it is not known to bind cofactors in Nqo1 (Sazanov and Hinchliffe, 2006). Although not discussed further here, there are additional differences in these two groups of proteins, which are present in all of the compared beta subunits (Supplementary File 1).

The above criteria were used to identify additional putative non-BF NADH-dependent hydrogenase sequences in our previously compiled database of hydrogenase sequences (Poudel et al., 2016). Using this database, 24 additional homologs were identified that are predicted to be non-BF NADH-dependent enzymes that were previously identified as group2 BF trimeric hydrogenases (Supplementary Table S1). Similarly, an additional non-BF NADH-dependent enzyme homolog was identified that was previously predicted to be a group3 BF tetrameric hydrogenase (Supplementary Table S2). These criteria also identified non-BF NADH-dependent [FeFe]-hydrogenases in the genomes of known syntrophic organisms (Supplementary Table S3) and in the hydrogenosomes of the anaerobic ciliate *Nyctotherus ovalis*. In the case of *N. ovalis*, this assignment is consistent with the proposal that this enzyme is a non-BF NADH-dependent enzyme (Akhmanova et al., 1998; Boxma et al., 2007; Supplementary Table S3).

A phylogenetic tree of beta subunit protein sequences associated with BF and non-BF NADH dependent enzymes recapitulates the differences identified above and demarcates known BF enzymes from non-BF enzymes. Moreover, seeding this phylogeny with 137 group2 trimeric and 72 group3 tetrameric [FeFe]-hydrogenases (Poudel et al., 2016) reveals that beta subunits from non-BF enzymes form a separate clade from the beta subunits of BF enzymes (Supplementary Figure S6).

## Discussion

The HydAB from *S. aciditrophicus* functions in a manner similar to the [FeFe]-hydrogenase of *S. wolfei*, Hyd1ABC (Losey et al., 2017). Both enzymes are non-BF, NADH-dependent and function without the involvement of ferredoxin to reversibly produce hydrogen from NADH. The lack of ferredoxin dependence by these enzymes may be an evolutionary outcome specific to syntrophy (i.e., growth at very low hydrogen partial pressures) as syntrophic organisms are known to require low hydrogen partial pressures for growth and metabolism (Sieber et al., 2012). The use of a non-BF NADH-dependent [FeFe]-hydrogenase would require continual hydrogen removal by a hydrogen-consuming partner. Thus, the use of a NADH dependent, non-BF hydrogenase explains, in part, the obligate requirement that *S. aciditrophicus* and *S. wolfei* have for a hydrogen-consuming partner (i.e., hydrogentrophic methanogens) during growth on substrates such as fatty and alicyclic acids.

The use of a non-BF [FeFe]-hydrogenase instead of a BF [FeFe]-hydrogenase is advantageous to syntrophic organisms as the syntroph does not have to use energy established in the form of an electrochemical gradient across the membrane to generate reduced ferredoxin for every NADH that is oxidized. Non-BF NADH-dependent hydrogen production is consistent with the known physiological properties of the syntrophic fatty, aromatic and alicyclic acid oxidizers, which use degradative pathways that do not generate reduced ferredoxin (McInerney et al., 2007; Sieber et al., 2010, 2015; James et al., 2019). In support of this concept, peptides matching ferredoxins were not detected in the proteome of *S. wolfei* (Sieber et al., 2015; Losey et al., 2017). While *S. aciditrophicus* encodes both ferredoxin and the ferredoxin-generating Rnf complex (McInerney et al., 2007), it is likely that Rnf in *S. aciditrophicus* functions to make reduced ferredoxin for benzoyl-CoA reduction and biosynthetic reactions (McInerney et al., 2007; Fuchs et al., 2011; Kung et al., 2013; James et al., 2019).

The subunit composition of *S. aciditrophicus* HydAB, α_2_β_2_ (Figure 3), differs from that of *S. wolfei* HydABC, αβγ (Losey et al., 2017). A BLASTP comparison showed 37% amino acid sequence identity between the *S. wolfei* gamma subunit (Hyd1C) and the N-terminal portion (amino acid residues 15–152) of the *S. aciditrophicus* beta subunit (HydB). Another BLASTP comparison showed 46% amino acid sequence identity between the *S. wolfei* beta subunit (Hyd1B) to the C-terminal portion of the *S. aciditrophicus* beta subunit (HydB) (amino acid residues 177–585). Thus, *S. aciditrophicus* beta subunit comprises a fusion of *S. wolfei* Hyd1BC. In this case, the fused gamma subunit-like region is at the N-terminal portion of the beta subunit (Figure 3). The monomeric *Nyctotherus ovalis* [FeFe]-hydrogenase, which was previously proposed to be non-BF and NADH-dependent (Akhmanova et al., 1998; Boxma et al., 2007), appears also to be a fused protein that includes the equivalent of the alpha, beta and gamma subunits of *S. wolfei* Hyd1ABC (Figure 3).

Comparison of known BF and non-BF NADH dependent enzymes allowed for the establishment of several criteria for delineating enzyme homologs (Figure 5). Applying these criteria, non-BF NADH-dependent [FeFe]-hydrogenases were identified in the genomes of known fatty acid-oxidizing syntrophic metabolizers (*Syntrophomonas zehnderi*, *Syntrophomonas wolfei* subsp. *methylbutyratica*, *Syntrophomonas palmitatica, Syntrophothermus lipocalidus*, *Thermosyntropha lipolytica*, and *Syntrophobotulus glycolicus*) (Supplementary Tables S1, S3). The syntrophic acetate oxidizer, *Syntrophaceticus schinkii*, also encodes a non-BF NADH-dependent [FeFe]-hydrogenase (Manzoor et al., 2016). However, the propionate-degrading syntrophic metabolizers, *Syntrophobacter fumaroxidans* and *Pelotomaculum thermopropionicum*, encode BF [FeFe]-hydrogenases (Supplementary Tables S1, S2). A possible explanation for this is that propionate degradation by the methyl-malonyl-CoA pathway (Visser et al., 2013; Sedano-Núñez et al., 2018) generates both NADH and reduced ferredoxin (from pyruvate metabolism) and the use of a BF [FeFe]-hydrogenase would allow the concurrent oxidation of both cofactors.

Many of the newly detected non-BF NADH-dependent [FeFe]-hydrogenases sequences were identified in the genomes of anaerobic genera not known to be syntrophic metabolizers such as *Ruminiclostridum*, *Clostridium*, *Spirochaeta*, *Dehalococcoides*, *Dehalobacter*, *Halothermothrix*, *Ignavibacterium*, *Desulfitobacterium*, *Selenomonoas*, *Halanaerobium, Marinitoga*, *Mahella*, *Petrotoga*, and *Candidatus Cloacimonas*. Some of the genomes are predicted to encode both non-BF and BF [FeFe]-hydrogenases (Supplementary Tables S1, S2), suggesting that anaerobes may use different [FeFe]-hydrogenases depending on prevailing hydrogen concentrations such as the different strategies employed by *Ruminococcus albus* (Zheng et al., 2014; Jay et al., 2020). The presence of non-BF NADH-dependent [FeFe]-hydrogenase in anaerobes not known to be syntrophic metabolizers suggests that non-BF [FeFe]-hydrogenase may have physiological functions that differ from facilitating syntrophic metabolism (Scheifinger et al., 1975; Asanuma and Hino, 2000; Mansfeldt et al., 2014; Zinder, 2016). Therefore, it is unlikely that the presence of a non-BF NADH-dependent [FeFe]-hydrogenase can be used as the sole indicator for the capability for syntrophic metabolism. Instead, such determinations will require additional considerations such as the presence/absence of metabolic pathways that generate reduced ferredoxin or provide alternate routes to oxidize reduced cofactors. For example, the *N. ovalis* [FeFe]-hydrogenase meets all the criteria that were identified to be delineated as a non-BF NADH-dependent [FeFe]-hydrogenase. In addition, the *N. ovalis* genome does not encode enzymes that are known to produce reduced ferredoxin such as pyruvate:ferredoxin oxidoreductase and thus would be unable to generate reduced ferredoxin needed for BF [FeFe]-hydrogenase activity (Boxma et al., 2007; de Graaf et al., 2011). Thus, *N. ovalis* appears to produce hydrogen solely from NADH, which would require a hydrogen-consuming partner in close proximity to the hydrogenosome to maintain a low hydrogen partial pressure. Indeed, there is a hydrogenotrophic methanogen, resembling *Methanobrevibacter*, in close association to the hydrogenosome organelle in *N. ovalis* and it may function to maintain low hydrogen partial pressures needed for hydrogen production from the non-BF NADH hydrogenase (Gijzen et al., 1991).

**FIGURE 6:**
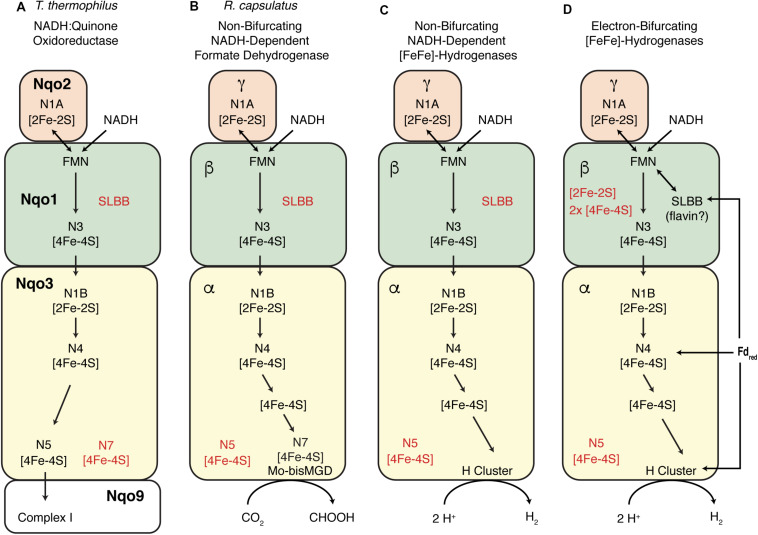
Comparison of electron flow pathways in NADH-dependent enzymes. **(A)** Representation of electron flow as proposed for the structurally solved *T. thermophilus* Nqo1-Nqo3 subunits as part of Respiratory Complex 1, with [Fe-S] centers numbering as described in Sazanov and Hinchliffe (2006). [Fe-S] centers that are equivalent to those in Nqo1-3 are indicated in other panels. The N1A cluster is proposed to help mediate the two electron transfer from NADH (Gnandt et al., 2016; Baymann et al., 2018). **(B)** Representation of the proposed electron flow for the metal-containing *R. capsulatus* formate dehydrogenase as proposed by Hille et al. (2014) which was based of homology modeling in the reference. **(C)** Proposed electron flow in non-BF NADH-dependent [FeFe]-hydrogenases reveals similarities with the *R. capsulatus* formate dehydrogenase with the exception of the [H]-cluster and the nearest [4Fe-4S] center which is not shared with the formate dehydrogenase. **(D)** Representation of several proposals for electron flow in multimeric BF [FeFe]-hydrogenases. It has been proposed that separate electron paths are required for electron flow from NADH and ferredoxin to a site of electron-bifurcation (Buckel and Thauer, 2018; Peters et al., 2018). The details of electron flow in BF [FeFe]-hydrogenases are unknown as are the locations and roles of the additional [Fe-S] centers present.

The high sequence homology (>39% sequence identity) between the non-BF NADH-dependent FDH of *Cupriavidus necator* (formerly *Ralstonia eutropha*) and NADH:quinone oxidoreductase subunits Nqo1-3 (Sazanov and Hinchliffe, 2006; Hille et al., 2014) allowed the construction of a homology model of the *C. necator* enzyme (Hille et al., 2014) based on the crystal structure of the *T. thermophilus* Nqo1-3 (Sazanov and Hinchliffe, 2006). A similar approach allowed for the recent construction of a partial model the *T. maritima* BF [FeFe]-hydrogenase (Chongdar et al., 2019). Given the homology between *S. aciditrophicus* [FeFe]-hydrogenase and the *C. necator* and *T. thermophilus* enzymes, it is possible that the electron flow path in non-BF NADH-dependent [FeFe]-hydrogenases is similar to those in the *T. thermophilus* and *C. necator* enzymes (Figure 6). For the non-BF NADH-dependent [FeFe]-hydrogenases, this implies that electrons pass from the NADH-binding site to the [H]-cluster catalytic site without involvement of a second (bifurcating) path involving ferredoxin. One can hypothesize that the flow path of electrons from NADH in BF [FeFe]-hydrogenases is the same as that in the non-BF NADH-dependent enzymes, but there must be an additional flow path for the electrons from reduced ferredoxin and an addition cofactor or metal cluster to mediate electron bifurcation (Baymann et al., 2018; Buckel and Thauer, 2018; Muller et al., 2018; Peters et al., 2018). The details of electron flow and the site of electron bifurcation in BF [FeFe]-hydrogenases are still unclear, with multiple proposals for the cofactor serving as the site of electron bifurcation including: (1) a loosely bound flavin cofactor in the beta subunit (Buckel and Thauer, 2018; Kpebe et al., 2018; Schuchmann et al., 2018), (2) the [H]-Cluster (Peters et al., 2018), or (3) an [4Fe-4S] cluster of the alpha subunit (Chongdar et al., 2019; Figure 6D). Our proposal provides a hypothesis to understand the path of electron flow from NADH to the active site of H_2_ production, the H-cluster (Figure 6). However, the details of electron flow from reduced ferredoxin and the mechanism of electron bifurcation in the BF [FeFe]-hydrogenases have yet be determined.

## Conclusion

The beta subunits of the *S. wolfei* and *S. aciditrophicus* [FeFe]-hydrogenases share features with the beta subunits of other non-BF NADH-dependent enzymes and these are distinct form the beta subunits of BF [FeFe]-hydrogenases. Four criteria were proposed to differentiate between the beta subunits of BF enzymes from those of non-BF NADH-dependent enzymes. The function of these conserved residues in BF enzymes, if any, is not known but they represent targets for further research. The criteria outlined herein to differentiate BF and non-BF [FeFe]-hydrogenase enzymes based on beta subunit characteristics, when applied to our database of homologs, identified additional multimeric [FeFe]-hydrogenase homologs that are predicated to be non-BF and NADH dependent. The known physiology of the some of these microorganisms suggests that non-BF NADH-dependent [FeFe]-hydrogenases may be involved in metabolic processes other than syntrophic fatty acid metabolism. However, a microorganism that produces hydrogen or formate from NADH in a ferredoxin-independent manner could be considered a syntrophic metabolizer because a hydrogen- or formate-using partner would be required to continually produce hydrogen or formate from NADH if no other route for NADH oxidation exits in the microorganism. The system for oxidizing reduced flavin cofactors (EtfAB) during syntrophic fatty acid metabolism also requires low hydrogen partial pressure (<60 Pa) (Wallrabenstein and Schink, 1994; Schmidt et al., 2013; Sieber et al., 2015; Crable et al., 2016) and microorganisms using such systems may also be considered syntrophic metabolizers.

## Data Availability Statement

The datasets analyzed for this study can be found in the Genbank Repository (see Supplementary Tables S1–S3 for accession numbers).

## Author Contributions

NL and MM participated in the design of the experiments and writing the manuscript. NL performed the experimentation and bioinformatic analyses. SP and EB curated the databases, performed the alignments, and participated in writing the manuscript.

## Conflict of Interest

The authors declare that the research was conducted in the absence of any commercial or financial relationships that could be construed as a potential conflict of interest.

## Abbreviations

BF: electron-bifurcating
Non-BF: non-bifurcating.
