# Supplementary material for: The Beta Subunit of Non-bifurcating NADH-Dependent [FeFe]-Hydrogenases Differs From Those of Multimeric Electron-Bifurcating [FeFe]-Hydrogenases
Source: Front Microbiol. 2020 Jun 17;11:1109. doi: 10.3389/fmicb.2020.01109 (PMC7311640; doi:10.3389/fmicb.2020.01109)
Supplement: Supplementary file 1 [file Data_Sheet_1.pdf]

Supplemental Tables for

The Beta Subunit of Non-Bifurcating NADH-Dependent [FeFe]-Hydrogenases Differ from Those of Multimeric Electron-Bifurcating [FeFe]-Hydrogenases

Nathaniel A. Losey, Saroj Poudel, Eric S. Boyd, and Michael J. McInerney

Table S1. Beta Subunits from Group 2 Trimeric [FeFe]-Hydrogenases. Sequences highlighted in blue have the criteria proposed for solely NADH-dependent, non-BF activity while those in black have the proposed criteria for BF activity. Sequences in orange have only 2 of 3 BF criteria.

Organism	Genbank ID	# of AA Residues	Residue at NADH site 1 (TMHydB residue #232)	Residue at NADH site 2 (TmHydB residue #234)	Residue at FMN site (~TmHydB residue #312)	Residues at GGPSG motif site (TmHydB residue #427-#431)
Spirochaeta thermophila DSM 6192	YP_003873857	418	T	K	Y	GGTAG
Halothermothrix orenii H 168	YP_002508053	408	T	K	Y	GGIST
Halanaerobium hydrogeniformans	YP_003995542	410	T	K	Y	GGVST
Marinitoga piezophila KA3	AEX85426	402	T	K	Y	GGIAT
Petrotoga mobilis SJ95	YP_001568191	404	T	K	Y	GGIST
Ignavibacterium album JCM 16511	YP_005845236	409	T	K	Y	GGASG
Syntrophobotulus glycolicus DSM 8271	YP_004267185	427	T	K	Y	GGSSG
Desulfitobacterium dehalogenans ATCC 51507	AFL99064	431	T	K	Y	GGSSG
Ruminiclostridium papyrosolvens DSM 2782	EGD46501	429	T	K	Y	GGSSG

Clostridium sp. BNL1100	AEY65021	429	T	K	Y	GGSSG
Selenomonas ruminantium subsp. *lactilytica* TAM6421	YP_005434366	416	T	K	Y	GGASG
Selenomonas sputigena	WP_006191643	416	T	K	Y	GGASG
Dehalobacter restrictus DSM 9455	AHF10703	425	T	K	Y	GGASG
Dehalobacter sp. DCA	AFV02665	425	T	K	Y	GGASG
Mahella australiensis 50-1 BON	AEE96575	434	T	K	Y	GGASG
Dehalococcoides mccartyi VS	YP_003329655	417	T	K	Y	GGISS
Dehalococcoides mccartyi GY50	AHB12962	417	T	K	Y	GGISS
Dehalococcoides mccartyi 195	YP_180896	417	T	K	Y	GGISS
Dehalococcoides mccartyi GT	YP_003461997	417	T	K	Y	GGISS
Dehalococcoides mccartyi BTF08	AGG07215	417	T	K	Y	GGISS
Dehalococcoides mccartyi CBDB1	YP_307337	417	T	K	Y	GGISS
Dehalococcoides mccartyi DCMB5	AGG05801	417	T	K	Y	GGISS
Dehalococcoides mccartyi BAV1	YP_001213692	417	T	K	Y	GGISS
Syntrophothermus lipocalidus DSM 12680	YP_003701489	407	T	K	Y	GGTSG
Sphaerochaeta pleomorpha str. Grapes	AEV28062	1050	A	M	F	GGPSG
Sphaerochaeta globosa str. Buddy	ADY13418	1050	A	M	F	GGPSG
Halobacteroides halobius DSM 5150	AGB40371	600	A	M	F	GGPSG

Acetohalobium arabaticum DSM 5501	YP_003828452	600	A	M	F	GGPSG
Halothermothrix orenii H 168	YP_002508384	594	A	M	F	GGPSG
Desulfotomaculum ruminis DSM 2154	YP_004544087	645	A	M	F	GGPSG
Desulfotomaculum acetoxidans DSM 771	YP_003193157	677	A	M	F	GGPSG
Pelotomaculum thermopropionicum SI	YP_001211928	650	A	M	F	GGPSG
Symbiobacterium thermophilum IAM 14863	YP_077034	618	A	M	F	GGPSG
Moorella thermoacetica ATCC 39073	YP_430563	619	A	M	F	GGPSG
Desulfotomaculum ruminis	YP_004545924	569	A	M	F	GGPSG
Desulfotomaculum nigrificans CO-1-SRB	AEF94085	569	A	M	F	GGPSG
Desulfotomaculum reducens MI-1	YP_001113005	569	A	M	F	GGPSG
Candidatus *Desulforudis audaxviator* MP104C	YP_001717479	572	A	M	F	GGPSG
Pelotomaculum thermopropionicum SI	YP_001212561	551	A	M	F	GGPSG
Syntrophobacter fumaroxidans MPOB	YP_844977	572	A	M	F	GGPSG
Dehalobacter restrictus DSM 9455	AHF10171	1028	A	M	F	GGPSG
Dehalobacter sp. DCA	AFV01902	1028	A	M	F	GGPSG
Spirochaeta africana DSM 8902	AFG37808	1063	A	M	F	GGPSG
Spirochaeta smaragdinae DSM 11293	YP_003802216	1040	A	M	F	GGPSG
Opitutus terrae PB90-1	YP_001818429	668	A	M	F	GGPSG

Turneriella parva DSM 21527	AFM13385	610	A	M	Y	GGPSG
Elusimicrobium minutum Pei191	ACC98087	620	A	M	F	GGPSG
Treponema primitia ZAS-2	YP_004529576	624	A	M	F	GGPSG
Brachyspira hyodysenteriae WA1	YP_002722660	562	A	M	F	GGPSG
Brachyspira intermedia PWS/A	AEM21356	562	A	M	F	GGPSG
Brachyspira murdochii DSM 12563	YP_003634434	562	A	M	F	GGPSG
Ruminococcus albus 7 DSM 20455	YP_004106065	630	A	M	F	GGPSG
Bacteroides thetaiotaomicron VPI-5482	NP_809038	635	A	M	F	GGPSG
Bacteroides xylanisolvens XB1A	CBK69049	635	A	M	F	GGPSG
Sebaldella termitidis ATCC 33386	YP_003307428	614	A	M	F	GGPSG
Sebaldella termitidis ATCC 33386	YP_003310365	614	A	M	F	GGPSG
Ilyobacter polytropus DSM 2926	YP_003966825	598	A	M	F	GGPSG
Ruminiclostridium papyrosolvens DSM 2782	EGD48368	575	A	M	F	GGPSG
Desulfotomaculum reducens MI-1	YP_001114616	575	A	M	F	GGPSG
Clostridium ljungdahlii DSM 13528	YP_003779641	626	A	M	F	GGPSG
Clostridium autoethanogenum DSM 10061	AGY77773	626	A	M	F	GGPSG

Clostridium botulinum A str. ATCC 3502	YP_001254349	631	A	M	F	GGPSG
Clostridium botulinum A str. ATCC 19397	YP_001384106	631	A	M	F	GGPSG
Clostridium botulinum A str. Hall	YP_001387646	631	A	M	F	GGPSG
Clostridium botulinum H04402 065	CBZ03652	631	A	M	F	GGPSG
Clostridium botulinum B1 str. Okra	YP_001781396	631	A	M	F	GGPSG
Clostridium botulinum A3 str. Loch Maree	YP_001787173	631	A	M	F	GGPSG
Clostridium botulinum F str. Langeland	YP_001391108	631	A	M	F	GGPSG
Clostridium botulinum F str. 230613	ADF99531	631	A	M	F	GGPSG
Clostridium botulinum A2 str. Kyoto	YP_002804183	631	A	M	F	GGPSG
Clostridium botulinum Ba4 str. 657	YP_002862804	631	A	M	F	GGPSG
Clostridium cellulovorans 743B	YP_003843747	630	A	M	F	GGPSG
Clostridium sticklandii	YP_003935938	625	A	M	F	GGPSG
Clostridioides difficile 630	YP_001089926	628	A	M	F	GGPSG
Peptoclostridium difficile CD196	YP_003216196	628	A	M	F	GGPSG
Peptoclostridium difficile	YP_003219703	628	A	M	F	GGPSG
Peptoclostridium difficile BI1	YP_006200432	628	A	M	F	GGPSG
Clostridiales sp.	WP_003416938	628	A	M	F	GGPSG
Desulfosporosinus acidiphilus SJ4	AFM39372	628	A	M	F	GGPSG

Desulfosporosinus orientis DSM 765	AET67216	628	A	M	F	GGPSG
Desulfosporosinus meridiei DSM 13257	AFQ42723	628	A	M	F	GGPSG
Desulfosporosinus acidiphilus SJ4	AFM43311	627	A	M	F	GGPSG
Desulfosporosinus meridiei DSM 13257	AFQ46172	627	A	M	F	GGPSG
Alkaliphilus oremlandii OhILAs	YP_001512472	635	A	M	F	GGPSG
Anaerococcus prevotii DSM 20548	YP_003152834	526	A	M	F	GGPSG
Clostridium ljungdahlii DSM 13528	YP_003778876	599	A	M	F	GGPSG
Clostridium autoethanogenum DSM 10061	AGY77002	599	A	M	F	GGPSG
Ruminiclostridium thermocellum ATCC 27405	YP_001036860	624	A	M	F	GGPSG
Clostridium lentocellum DSM 5427	YP_004308500	624	A	M	F	GGPSG
Clostridium saccharoperbutylacetonicum N1-4(HMT)	YP_007457382	624	A	M	F	GGPSG
Clostridium autoethanogenum DSM 10061	AGY75800	624	A	M	F	GGPSG
Treponema primitia ZAS-2	YP_004529858	627	A	M	F	GGPSG
Treponema primitia ZAS-2	YP_004529844	634	A	M	F	GGPSG
Ruminococcus sp. 5 1 39BFAA	EES78091	623	A	M	F	GGPSG
Ruminococcus sp. SR1/5	CBL19413	623	A	M	F	GGPSG
Blautia obeum A2-162	CBL24111	623	A	M	F	GGPSG

Ruminococcus torques L2-14	CBL25630	622	A	M	F	GGPSG
Ruminiclostridium papyrosolvens DSM 2782	EGD49694	623	A	M	F	GGPSG
Clostridium sp. BNL1100	AEY66910	623	A	M	F	GGPSG
Clostridium cellulolyticum H10	YP_002506621	623	A	M	F	GGPSG
Ethanoligenens harbinense YUAN-3	YP_004092839	624	A	M	F	GGPSG
Alkaliphilus oremlandii OhILAs	YP_001512532	623	A	M	F	GGPSG
Lachnoclostridium phytofermentans ISDg	YP_001557217	628	A	M	F	GGPSG
Clostridium beijerinckii NCIMB 8052	YP_001311177	626	A	M	F	GGPSG
Clostridium botulinum B str. Eklund 17B (NRP)	YP_001886497	626	A	M	F	GGPSG
Clostridium botulinum E3 str. Alaska E43	YP_001921432	626	A	M	F	GGPSG
Oscillibacter valericigenes Sjm18-20	BAK98861	629	A	M	F	GGPSG
Fretibacterium fastidiosum	CBL28840	628	A	M	F	GGPSG
Thermosediminibacter oceani DSM 16646	YP_003824933	625	A	M	F	GGPSG
Tepidanaerobacter acetatoxydans Re1	YP_004461912	625	A	M	F	GGPSG
Desulfobulbus propionicus DSM 2032	YP_004195043	633	A	M	F	GGPSG
Pelobacter propionicus DSM 2379	YP_901163	570	A	M	F	GGPSG
Desulfovibrio magneticus RS-1	YP_002952161	629	A	M	F	GGPSG

Spirochaeta smaragdinae DSM 11293	YP_003802104	632	A	M	F	GGPSG
Melioribacter roseus P3M-2	AFN73878	634	A	M	F	GGPSG
Halothermothrix orenii H 168	YP_002507937	624	A	M	F	GGPSG
Desulfotomaculum ruminis DSM 2154	YP_004545920	626	A	M	F	GGPSG
Desulfotomaculum reducens MI-1	YP_001113008	627	A	M	F	GGPSG
Desulfotomaculum nigrificans CO-1-SRB	AEF94091	627	A	M	F	GGPSG
Desulfotomaculum kuznetsovii DSM 6115	YP_004518296	626	A	M	F	GGPSG
Desulfallas gibsoniae DSM 7213	AGL02551	626	A	M	F	GGPSG
Fervidobacterium pennivorans DSM 9078	AFG34975	634	A	M	F	GGPSG
Fervidobacterium nodosum Rt17-B1	YP_001409750	632	A	M	F	GGPSG
Pseudothermotoga thermarum DSM 5069	AEH50204	625	A	M	F	GGPSG
Thermosipho africanus TCF52B	YP_002334589	624	A	M	F	GGPSG
Thermosipho melanesiensis BI429	YP_001305743	623	A	M	F	GGPSG
Dictyoglomus thermophilum H-6-12	YP_002250293	624	A	M	F	GGPSG
Dictyoglomus turgidum DSM 6724	YP_002352463	624	A	M	F	GGPSG
Pseudothermotoga elfii	WP_028843228	626	A	M	F	GGPSG
Thermotoga lettingae TMO	YP_001470583	626	A	M	F	GGPSG
Thermotoga maritima MSB8	NP_229225	626	A	M	F	GGPSG

Thermotoga sp. RQ2	YP_001739345	626	A	M	F	GGPSG
Thermotoga petrophila RKU-1	YP_001244958	626	A	M	F	GGPSG
Thermotoga naphthophila RKU-10	YP_003346883	626	A	M	F	GGPSR
Thermotoga neapolitana DSM 4359	YP_002534610	626	A	M	F	GGPSG
Melioribacter roseus P3M-2	AFN75711	661	A	M	F	GGPSG
Ignavibacterium album JCM 16511	YP_005845510	661	A	M	F	GGPSG

Table S2. Beta Subunits from Group 3 Tetrameric [FeFe]-Hydrogenases.
Sequences highlighted in blue have the criteria proposed for non-BF NADH-dependent activity while those in black have the criteria for BF activity.

Organism	Genbank ID	# of AA Residues	Residue at NADH site 1 (TMHydB residue #232)	Residue at NADH site 2 (TmHydB residue #234)	Residue at FMN site (~TmHydB residue #312)	Residues at GGPSG motif site (TmHydB residue #427-#431)
Candidatus Cloacimonas acidaminovorans str. Evry	YP_001741249	520	T	K	Y	GGAAG
Acetobacterium woodii DSM 1030	AFA49451	599	A	M	F	GGPSG
Caldicellulosiruptor obsidiansis OB47	YP_003840537	598	A	M	F	GGPSG
Caldicellulosiruptor hydrothermalis 108	YP_003992514	598	A	M	F	GGPSG
Caldicellulosiruptor kronotskyensis 2002	YP_004024078	598	A	M	F	GGPSG
Clostridium sp. SY8519	YP_004709436	595	A	M	F	GGPSG
Halanaerobium hydrogeniformans	YP_003995635	600	A	M	F	GGPSG
Halanaerobium praevalens DSM 2228	ADO77754	601	A	M	F	GGPSG
Spirochaeta thermophila DSM 6578	AEJ61771	595	A	M	F	GGPSG
Thermoanaerobacter sp. X514	YP_001663747	596	A	M	F	GGPSG
Thermoanaerobacter pseudethanolicus ATCC 33223	YP_001665443	596	A	M	F	GGPSG
Thermoanaerobacter brockii subsp. *finnii* Ako-1	YP_004186441	596	A	M	F	GGPSG
Tepidanaerobacter acetatoxydans Re1	YP_004461325	597	A	M	F	GGPSG

Thermoanaerobacterium xylanolyticum LX-11	YP_004471284	596	A	M	F	GGPSG
Thermoanaerobacterium saccharolyticum JW/SL-YS485	AFK87131	596	A	M	F	GGPSG
Treponema caldarium DSM 7334	AEJ20870	596	A	M	F	GGPSG
Spirochaeta thermophila DSM 6192	YP_003874588	595	A	M	F	GGPSG
Clostridium cellulolyticum H10	YP_002506555	597	A	M	F	GGPSG
Clostridium sp. BNL1100	AEY66838	597	A	M	F	GGPSG
Halobacteroides halobius DSM 5150	AGB42160	600	A	M	F	GGPSG
Spirochaeta smaragdinae DSM 11293	YP_003805368	595	A	M	F	GGPSG
Sphaerochaeta globosa str. Buddy	ADY14132	595	A	M	F	GGPSG
Treponema azotonutricium ZAS-9	YP_004528210	601	A	M	F	GGPSG
Desulfotomaculum acetoxidans DSM 771	YP_003189751	597	A	M	F	GGPSG
Desulfotomaculum kuznetsovii DSM 6115	YP_004517645	597	A	M	F	GGPSG
Dethiobacter alkaliphilus AHT 1	EEG77317	597	A	M	F	GGPSG
Alkaliphilus metalliredigens QYMF	YP_001321848	596	A	M	F	GGPSG
Marinitoga piezophila KA3	AEX86275	596	A	M	F	GGPSG
Dehalobacter restrictus DSM 9455	AHF10653	443	A	M	F	GGPSG
Dehalobacter sp. DCA	AFV02616	443	A	M	F	GGPSG

Pelobacter carbinolicus DSM 2380	YP_006717363	486	A	M	F	GGPSG
Petrotoga mobilis SJ95	YP_001567771	485	A	M	F	GGPSG
Clostridium lentocellum DSM 5427	YP_004309300	595	A	M	F	GGPSG
Odoribacter splanchnicus DSM 20712	YP_004251474	596	A	M	F	GGPSG
Caldanaerobacter subterraneus subsp. *tengcongensis* MB4	NP_622545	596	A	M	F	GGPSG
Caldicellulosiruptor saccharolyticus DSM 8903	YP_001180639	598	A	M	F	GGPSG
Caldicellulosiruptor bescii DSM 6725	YP_002573171	598	A	M	F	GGPSG
Caldicellulosiruptor owensensis OL	YP_004002452	598	A	M	F	GGPSG
Clostridium clariflavum DSM 19732	YP_005045888	597	A	M	F	GGPSG
Caldicellulosiruptor kristjanssonii I77R1B	YP_004026403	598	A	M	F	GGPSG
Caldicellulosiruptor lactoaceticus 6A	AEM73338	598	A	M	F	GGPSG
Ruminiclostridium thermocellum ATCC 27405	YP_001036772	597	A	M	F	GGPSG
Lachnoclostridium phytofermentans ISDg	YP_001560890	595	A	M	F	GGPSG
Coprothermobacter proteolyticus DSM 5265	YP_002246550	596	A	M	F	GGPSG
Clostridium saccharolyticum WM1	YP_003823543	595	A	M	F	GGPSG

Hungateiclostridium thermocellum	WP_003512583	597	A	M	F	GGPSG
Ruminococcus champanellensis 18P13 JCM 17042	CBL17695	597	A	M	F	GGPSG
Spirochaeta africana DSM 8902	AFG38439	598	A	M	F	GGPSG
Salinispira pacifica	AHC13717	594	A	M	F	GGPSG
Eubacterium limosum KIST612	YP_003958823	599	A	M	F	GGPSG
Thermoanaerobacter italicus Ab9	YP_003476669	596	A	M	F	GGPSG
Thermoanaerobacter mathranii subsp. *mathranii* str. A3	YP_003676615	596	A	M	F	GGPSG
Thermoanaerobacterium thermosaccharolyticum DSM 571	YP_003851723	596	A	M	F	GGPSG
Thermoanaerobacter sp. X513	YP_003903717	596	A	M	F	GGPSG
Thermoanaerobacter wiegelii Rt8.B1	AEM78408	596	A	M	F	GGPSG
Thermoanaerobacterium thermosaccharolyticum M0795	YP_007298420	596	A	M	F	GGPSG
Mahella australiensis 50-1 BON	AEE96447	597	A	M	F	GGPSG
Treponema brennaborense DSM 12168	YP_004438911	593	A	M	F	GGPSG
Thermosediminibacter oceani DSM 16646	YP_003825088	597	A	M	F	GGPSG
Sphaerochaeta pleomorpha str. Grapes	AEV30239	595	A	M	F	GGPSG

Ruminiclostridium papyrosolvens DSM 2782	EGD49783	597	A	M	F	GGPSG
Thermoclostridium stercorarium subsp. *stercorarium* DSM 8532	AGC67941	597	A	M	F	GGPSG
Alkaliphilus metalliredigens QYMF	YP_001318524	547	A	M	F	GGPSG
Acetohalobium arabaticum DSM 5501	YP_003826884	600	A	M	F	GGPSG
Desulfovibrio magneticus RS-1	YP_002951624	490	A	M	F	GGPSG
Geobacter sp. M18	YP_004198597	489	A	M	F	GGPSG
Pelobacter carbinolicus DSM 2380	YP_006717332	486	A	M	F	GGPSG
Thermanaerovibrio acidaminovorans DSM 6589	YP_003316671	596	A	M	F	GGPSG
Treponema pedis str. T A4	AGT43414	586	A	M	F	GGPSG
Parabacteroides distasonis ATCC 8503	YP_001302431	596	A	M	F	GGPSG
Desulfobacterium autotrophicum HRM2	YP_002602930	595	A	M	F	GGPSG
Desulfarculus baarsii DSM 2075	YP_003807481	596	A	M	F	GGPSG

Table S3. Non-BF Beta Subunits from Genomes of Selected Syntrophic Metabolizers and from *Nyctotherus ovalis*.

Organism	Genbank ID	# of AA Residues	Residue at NADH site 1 (TMHydB residue #232)	Residue at NADH site 2 (TmHydB residue #234)	Residue at FMN site (~TmHydB residue #312)	Residues at GGPSG motif site (TmHydB residue #427-#431)
Nyctotherus ovalis	AAU14235	1198	T	K	Y	GGCTE
Syntrophaceticus schinkii	WP_044664703	402	T	K	Y	GGNSG
Syntrophomonas zehnderi OL-4	WP_046500148	407	T	K	Y	GGTSG
Thermosyntropha lipolytica DSM 11003	WP_073091112	407	T	K	Y	GGTSG
Syntrophothermus lipocalidus DSM 12680	WP_013174328	407	T	K	Y	GGTSG
Syntrophomonas wolfei subsp. *methylbutyratica* JCM 14075	WP_061213711	407	T	K	Y	GGTSG
Syntrophomonas palmitatica JCM 14374	WP_054698037	407	T	K	Y	GGTSG

Table S4. Purification of [FeFe]-Hydrogenase, HydAB.

Fraction	Protein (mg)	Specific Activity* $H_2 \rightarrow MV_{ox}$ (U/mg)	Total Activity* $H_2 \rightarrow MV_{ox}$ (U)
Cell-free extract	194	0.36	69.8
HydAB Fraction	3.3	13	42.9

HydAB activity was followed by methyl viologen reduction with hydrogen as the electron donor at 37 °C and a pH of 7.5. One unit of activity (U) equals 2 μmoles of electrons transferred per min.

Table S5. Peptides Detected by HPLC MS-MS Analysis of the Purified Recombinant HydAB Fraction.

SwissProt ID	Protein Description	Gene Name	Number of Matches to Gene Product	Protein Sequence Coverage (%)
Q2LSB7	Iron only hydrogenase large subunit *Syntrophus aciditrophicus* (strain SB)	SYN_01370	805	81.16
Q2LSB6	NADH-quinone oxidoreductase chain F *Syntrophus aciditrophicus* (strain SB)	SYN_01369	840	65.07
P0AG44	50S ribosomal protein L17 *Escherichia coli* (strain K12)	rplQ	83	40.16
P77398	Bifunctional polymyxin resistance protein ArnA *Escherichia coli* (strain K12)	arnA	195	36.67
P0A9A9	Ferric uptake regulation protein *Escherichia coli* (strain K12)	fur	56	41.22
P0ACJ8	cAMP-activated global transcriptional regulator CRP *Escherichia coli* (strain K12)	crp	107	66.67
P17169	Glutamine--fructose-6-phosphate aminotransferase [isomerizing] *Escherichia coli* (strain K12)	glmS	191	48.77
P0A9K9	FKBP-type peptidyl-prolyl cis-trans isomerase SlyD *Escherichia coli* (strain K12)	slyD	109	47.96
P0A7S9	30S ribosomal protein S13 *Escherichia coli* (strain K12)	rpsM	36	49.15

P00393	NADH dehydrogenase *Escherichia coli* (strain K12)	ndh	89	35.94
Q2LS97	NADH-quinone oxidoreductase chain F *Syntrophus aciditrophicus* (strain SB)	SYN_02139	90	12.7
P0CE47	Elongation factor Tu 1 *Escherichia coli* (strain K12)	tufA	69	38.83
P0CE48	Elongation factor Tu 2 *Escherichia coli* (strain K12)	tufB	67	36.04
P0A951	Spermidine N(1)-acetyltransferase *Escherichia coli* (strain K12)	speG	31	50.54
P0A7V0	30S ribosomal protein S2 Escherichia coli (strain K12)	rpsB	56	30.71

Table S6. Peptides Detected by HPLC MS-MS Analysis of of Partially Purified *S. aciditrophicus* Ferredoxin Fraction.

SwissProt ID	Protein Description	Gene Name	Number of Matches to Gene Product	Protein Sequence Coverage (%)
Q2LUB2_SYNAS	Ferredoxin *Syntrophus aciditrophicus* (strain SB)	SYN_03059	24	98.31
Q2LR25_SYNAS	DNA-binding protein HU *Syntrophus aciditrophicus* (strain SB)	SYN_01724	35	27.47
ACP_SYNAS	Acyl carrier protein *Syntrophus aciditrophicus* (strain SB)	acpP	12	11.25
Q2LU87_SYNAS	Hypothetical cytosolic protein *Syntrophus aciditrophicus* (strain SB)	SYN_02004	11	13.98
Q2LSB7_SYNAS	Iron only hydrogenase large subunit *Syntrophus aciditrophicus* (strain SB)	SYN_01370	59	21.32
Q2LWS6_SYNAS	Glycine cleavage system H protein *Syntrophus aciditrophicus* (strain SB	gcvH	22	16.28
Q2LSR6_SYNAS	Integration host factor beta-subunit *Syntrophus aciditrophicus* (strain SB)	SYN_03200	9	9.68
Q2LX26_SYNAS	Molybdenum transport regulatory protein_ lysR family *Syntrophus aciditrophicus* (strain SB)	SYN_01042	9	20.66

COAD_SYNAS	Phosphopantetheine adenylyltransferase *Syntrophus aciditrophicus* (strain SB)	coaD	15	13.33
Q2LUY8_SYNAS	Acyl-CoA dehydrogenase_ short-chain specific *Syntrophus aciditrophicus* (strain SB)	SYN_00480	10	10.7
Q2LXH8_SYNAS	ABC-type nitrate/sulfonate/bicarbonate transport systems_ periplasmic components *Syntrophus aciditrophicus* (strain SB)	SYN_00365	15	10.33
Q2LUY7_SYNAS	Glutaconyl-CoA decarboxylase A subunit *Syntrophus aciditrophicus* (strain SB)	SYN_00481	15	7.97
Q2LWR8_SYNAS	Acetyl-CoA synthetase *Syntrophus aciditrophicus* (strain SB)	SYN_02635	20	9.91

Table S7. Comparison of Cofactor-Binding Sites between BF [FeFe]-Hydrogenases and Formate Dehydrogenases and Homologous Non-BF NADH-Dependent Enzymes.

Data are from cited references except in the case for the enzymes from *C. oxalaticus, M. trichosporum, M. extorquens, E. coli*, and *T. thermophilus* where conserved domains were from predictions obtained from the InterPro database.

SLBB domain is indicated in all listed enzymes, even if it was not noted in cited references. For molecular weights, the term sequence indicates that the molecular weight for each subunit was predicted from deduced amino acid sequence while SDS-PAGE indicates that the molecular weight was obtained from SDS-PAGE analysis. Number of iron-sulfur clusters is indicated by the number of clusters followed by "x" in front of the cluster designation.

Source Organism	Literature Reference(s)	Protein Name	Alpha Subunit (Sequence Cofactors, Size)	Beta Subunit (Sequence Cofactors, Size)	Gamma Subunit (Sequence Cofactor, Size)	Additional Subunits (Sequence Cofactors, Size)	Genbank ID
Electron-Bifurcating (BF) Ferredoxin-Dependent, NADH-Dependent							
T. maritima	Verhagen et al. 1999, Schut and Adams 2009	HydABC	HydA : 3 x [4Fe4S], 2 x [2Fe2S], 1 x [H] : 72.3 kDa (Sequence)	HydB : 3 x [4Fe4S], 1 x [2Fe2S], FMN, NADH, SLBB 68.7 kDa (Sequence)	HydC : 1 x [2Fe2S] 18.0 kDa (Sequence)		AAD36496 AAD36495 AAD36494
A. woodii	Schuchmann and Muller 2012	HydABCD	HydA : 3 x [4Fe4S], 1 x [2Fe2S], 1 x [H] : 63.6 kDa (Sequence)	HydB : 3 x [4Fe4S], 1 x [2Fe2S], FMN, NADH, SLBB : 64.5 kDa (Sequence)	HydC : 1 X [2Fe2S] 14.1 kDa (Sequence)	HydD : No Cofactors : 14.3 kDa (Sequenc)	AFA49450 AFA49451 AFA49452 AFA49454
M. thermoacetica	Wang et al. 2013	HydABC	HydA : 3 x [4Fe4S], 1 x [2Fe2S], 1 x	HydB : 3 x [4Fe4S], FMN, NADH,	HydC : 1 X [2Fe2S] :		ABC20019 ABC20020 ABC20021

			[H] : 67 kDa (Sequence)	SLBB : 63 kDa (Sequence)	17 kDa (Sequence)		
R. albus	Zheng et al. 2014	HydABC	HydA : 3 x [4Fe4S], 1 x [2Fe2S], 1 x [H] : 64 kDa	HydB : 3 x [4Fe4S], 1 x [2Fe2S], FMN, NADH, SLBB : 68 kDa	HydC : 1 X [2Fe2S] : 18 kDa		ADU23430 ADU23431 ADU23432
C. tengcongenensis	Soboh et al. 2004	HydABCD	HydA : 3 x [4Fe4S], 1 x [2Fe2S], 1 x [H] : 64 kDa (SDS-PAGE)	HydB : 3 x [4Fe4S], 1 x [2Fe2S], FMN, NADH, SLBB : 65 kDa (SDS-PAGE)	HydC : 1 X [2Fe2S] : 20 kDa (SDS-PAGE)	HydD: No Cofactors : 14.1 kDa (SDS-PAGE)	AAM24150 AAM24149 AAM24148
C. acidurici	Wang et al. 2013	HylABC FdhF2	FdhF2 : 4 x [4Fe4S], 1 x [2Fe2S], 1 x Mo Sec : 99.0 kDa (Sequence)	HylB : 3 x [4Fe4S], 1 x [2Fe2S], FMN, NADH, SLBB : 68.0 kDa (Sequence)	HylC : : 1 X [2Fe2S] : 18.4 kDa (Sequence)	HylA : 4 x [4Fe4S], 2 x [2Fe2S] : 35.9 kDa (Sequence)	AFS79904 AFS79905 AFS79906 AFS79907
D. fructosovorans	Kpebe et al. 2018	HndABCD	HndD : 3 x [4Fe4S], 1 x [2Fe2S], 1 x [H] : 64 kDa (SDS-PAGE)	HndC : 3 x [4Fe4S], FMN, NADH, SLBB (Flavin) : 57 kDa (SDS-PAGE)	HndA : 1 X [2Fe2S] : 19 kDa (SDS-PAGE)	HndB : 1 X [2Fe2S] : 14 kDa (SDS-PAGE)	AAA87057 AAA87056 AAA87054 AAA87055
C. autoethangeneu m	Wang et al. 2013	FdhA HytABCD HytE12	FdhA : 1 x [4Fe4S], 1 x Mo Sec :	HytB : 3 x [4Fe4S], 1 x [2Fe2S], FMN, NADH,	HytC: : 1 X [2Fe2S] :18.1 kDa (Sequence)	HytA: 51.4 kDa (Sequence) HytC: 18.1	AGT29713 AGT29710 AGT29709 AGT29711

			78.8 kDa (Sequence)	SLBB : 65.5 kDa (Sequence)		kDa (Sequence) HytD : 28.6 kDa (Sequence) HytE1 : 19.9 kDa (Sequence) HytE2: 20.1 kDa (Sequence)	AGT29712 AGT29714 AGT29705
Not Bifurcating (Non-BF) NADH-Dependent							
S. wolfei	Losey et al. 2017	Hyd1ABC	HydA : 3 x [4Fe4S], 1 x [2Fe2S], 1 x [H] : 63.0 kDa (Sequence)	HydB : 1 x [4Fe4S], FMN, NADH, SLBB (Flavin) : 43.9 kDa (Sequence)	HydC : 1 x [2Fe2S] : 17.5 kDa (Sequence)		ABI68331 ABI68332 ABI68333
S. aciditrophicus	This work	HydAB	HydA : 3 x [4Fe4S], 1 x [2Fe2S], 1 x [H] : 68.5 kDa (Sequence)	HydB : 1 x [4Fe4S], 1 x [2Fe2S] FMN, NADH, SLBB (Flavin) : 66.0 kDa	Fused as N-terminal portion of HydB		ABC76974 ABC76975
R. capsulatus	Hartmann and Leimkuhler 2013	FdsABG	FdsA : 4 x [4Fe4S], 1 x [2Fe2S], 1 x Mo bis-MGD : 105	FdsB : 1 x [4Fe4S], FMN, NADH, SLBB (Flavin) : 45.0 kDa	FdsG: 1 x [2Fe2S] : 18.4 kDa (Sequence)		ADE86759 ADE86760 ADE86761

			kDa (Sequence)				
C. eutropha	Friedebold and Bowien 1993, Oh and Bowien 1998	FdhABG	FdhA : 4 x [4Fe4S], 1 x [2Fe2S], 1 x Mo bis-MGD : 110.0 kDa (SDS-PAGE)	FdhB : 1 x [4Fe4S], FMN, NADH, SLBB (Flavin) : 57.0 kDa (SDS-PAGE)	FdhG: 1 x [2Fe2S] : 11.6 kDa (SDS-PAGE)		WP_011614623 WP_011614622 WP_011614621 WP_010811246
C. oxalaticus	Muller et al. 1977	FdhABG (CDs from seq)	FdhA : 4 x [4Fe4S], 1 x [2Fe2S], 1 x Mo bis-MGD :	FdhB : 1 x [4Fe4S], FMN, NADH, SLBB (Flavin) :	FdhG: 1 x [2Fe2S] :		WP_063236918 WP_063236917 WP_063236916 WP_063236919
M. trichosporum	Jollie and Lipscomb 1993,	FdhABGD (CDs from seq)	FdhA : 4 x [4Fe4S], 1 x [2Fe2S], 1 x Mo bis-MGD : 98 kDa (SDS-PAGE)	FdhB : 1 x [4Fe4S], FMN, NADH, SLBB (Flavin) : 56 kDa (SDS-PAGE)	FdhG: 1 x [2Fe2S] : 20 kDa (SDS-PAGE)	FdhD : 11.5 kDa (SDS-PAGE)	WP_003614381 WP_003614380 WP_003614379 WP_003613007
M. extorquens	Laukel et al. 2003	Fdh1AB (CDs from seq)	FdhA : 4 x [4Fe4S], 1 x [2Fe2S], 1 x W bis-MGD : 105 kDa (SDS-PAGE)	FdhB : 1 x [4Fe4S], 1 x [2Fe2S] FMN, NADH, SLBB (Flavin) : 60 kDa (SDS-PAGE)	Fused as N-terminal portion of FdhB		ACS42636 ACS42635
E. coli		NuoEFG (CDs from seq)	NuoG : 3 x [4Fe4S], 1 x [2Fe2S] :	NuoF : 1 x [4Fe4S], FMN, NADH,	NuoE: 1 x [2Fe2S] :		NP_416786 NP_416787 NP_416788

T. thermophilus	Nqo123 (CDs from seq)	Nqo1 : 3 x [4Fe4S], 1 x [2Fe2S] :	SLBB (Flavin) : Nqo2 : 1 x [4Fe4S], FMN, NADH, SLBB (Flavin) :	Nqo3: 1 x [2Fe2S] :	AAA97943 AAA97942 AAA97944
