# Supplementary material for: The Beta Subunit of Non-bifurcating NADH-Dependent [FeFe]-Hydrogenases Differs From Those of Multimeric Electron-Bifurcating [FeFe]-Hydrogenases
Source: Front Microbiol. 2020 Jun 17;11:1109. doi: 10.3389/fmicb.2020.01109 (PMC7311640; doi:10.3389/fmicb.2020.01109)

Supplementary Figures for

The Beta Subunit of Non-Bifurcating NADH-Dependent [FeFe]-Hydrogenases Differ from Those of Multimeric Electron-Bifurcating [FeFe]-Hydrogenases

Nathaniel A. Losey, Saroj Poudel, Eric S. Boyd, and Michael J. McInerney

Supplementary Figure S1. SDS-PAGE Gel of Recombinant *S. aciditrophicus* HydAB Purified from *E. coli*. Lane 1, molecular weight markers; Lane 2, *E. coli* cell-free extract; Lane 3, HydAB fraction. Molecular weights listed on gel image were predicted from the encoding gene sequence.

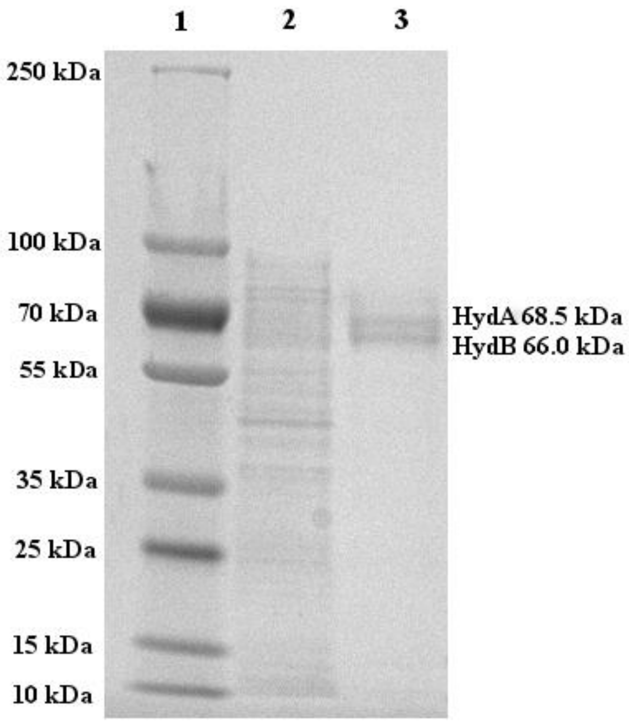

Supplementary Figure S2. Native PAGE Analysis of Recombinant *S. aciditrophicus* HydAB Purified from *E. coli*. Lane 1, molecular weight markers; Lane 2, *E. coli* cell-free extract; Lane 3, HydAB fraction.

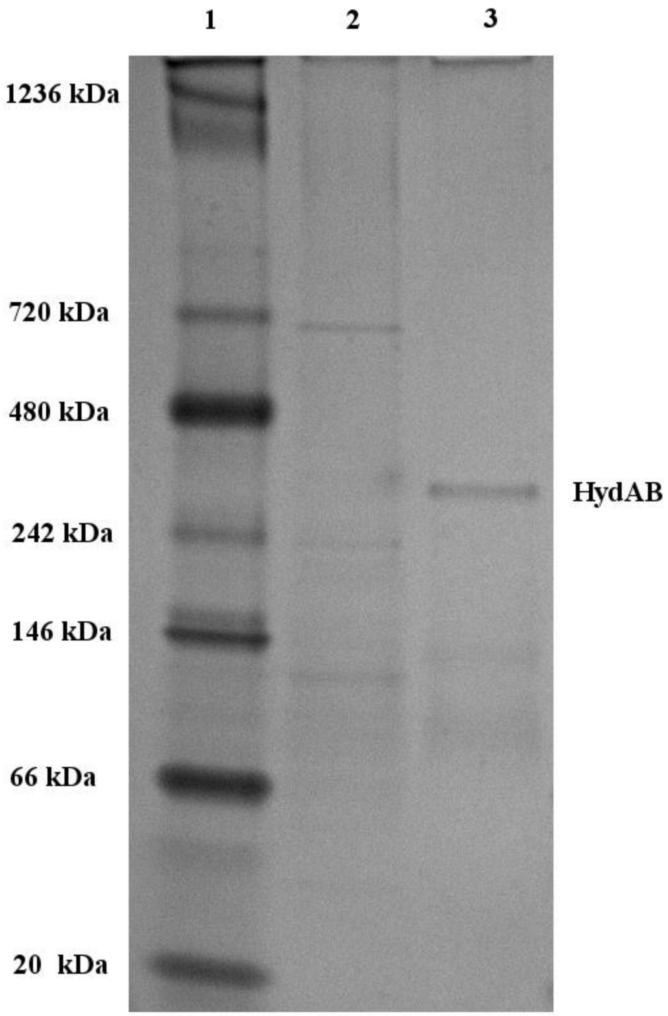

Supplementary Figure S3. SDS-PAGE of Recombinantly Produced *S. aciditrophicus* Ferredoxin from *E. coli* and Ferredoxin Partially Purified from *S. aciditrophicus*. Lane 1. molecular weight markers; Lane 2. nickel affinity purified recombinant SYN_03059 gene product; Lane 3. partially purified ferredoxin fraction from *S. aciditrophicus* cells.

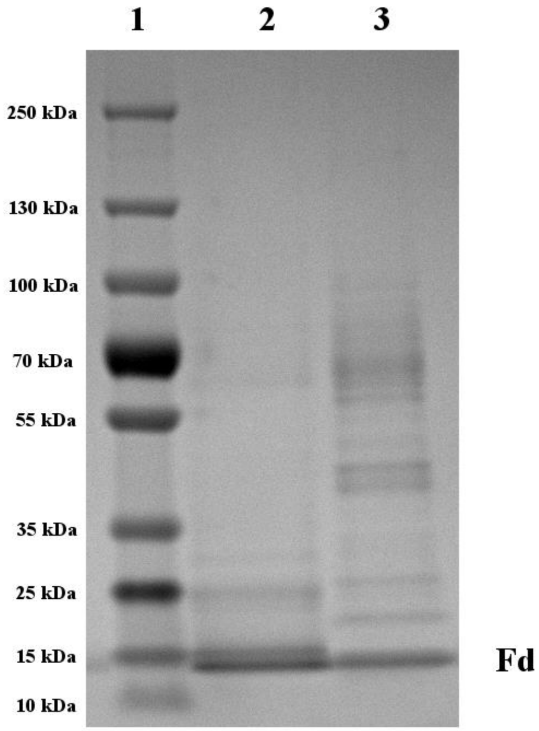

Supplementary Figure S4. Production of Hydrogen from NADH by HydAB in the Presence of Reduced Ferredoxin-Generating System. The reduced ferredoxin-generating system and HydAB (25.4 µg) were added as indicated by Fd_{red} and $+H_{ydAB}$, respectively. NADH was added as indicated by +NADH. The maximal rate of hydrogen production was 24.6 nanomoles • min^{-1}• mg^{-1}.

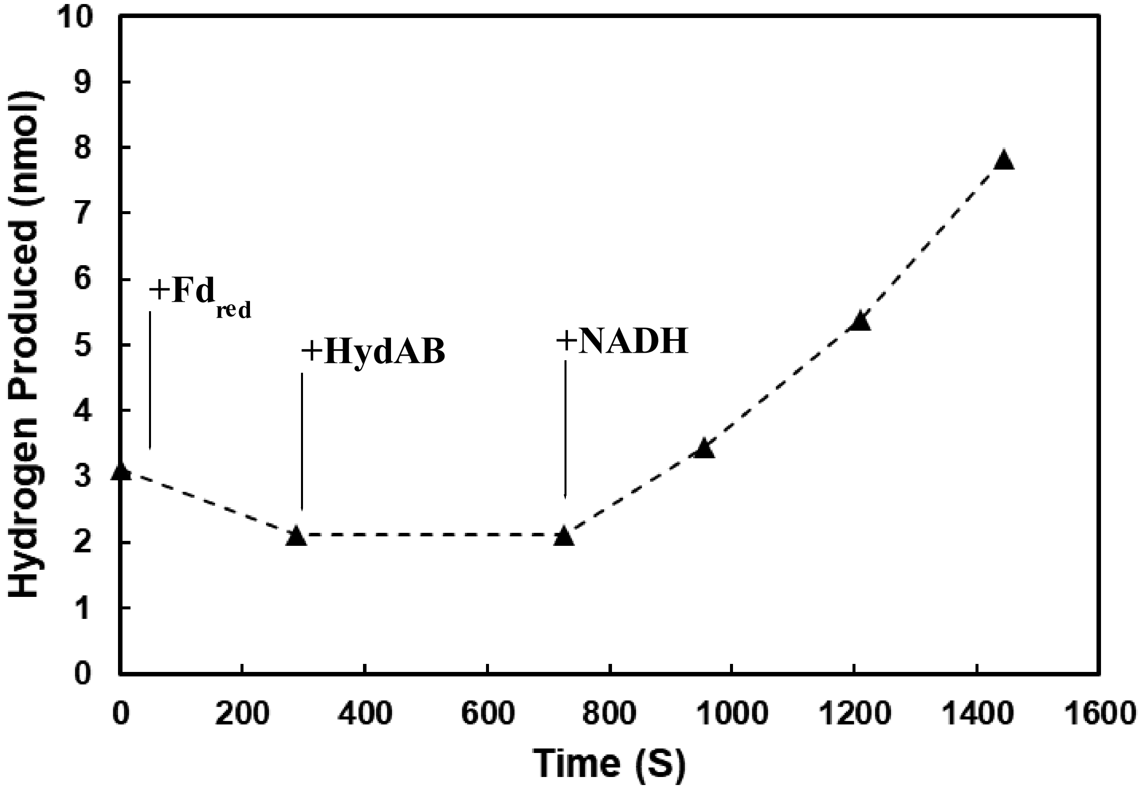

Supplementary Figure S5. Hydrogen Partial Pressures During the Metabolism of Crotonate, Cyclohex-1-ene-1-Carboxylate, and Benzoate By Pure Cultures of *S. aciditrophicus* and Co-Cultures of *S. aciditrophicus* with *Methanospirillum hungatei*. Panels A, B and C: hydrogen and substrate concentrations for metabolism of crotonate (A), cyclohex-1-ene carboxylate (B), and benzoate (C) by pure cultures of *S. aciditrophicus*. Panels D, E, and F: hydrogen and substrate concentrations during the metabolism of crotonate (D), cyclohex-1-ene carboxylate (E), and benzoate (F) by co-cultures of *S. aciditrophicus* with *M. hungatei*. (A) 97.8 ±9.8 nmol, (B) 127.5 ± 6.9 nmol, (C) 72.2 ± 9.8 nmol, (D) 57.4 ± 9.1 nmol, (E) 56.8 ± 7.3 nmol, (F) 46.9 ± 28.8 nmol.

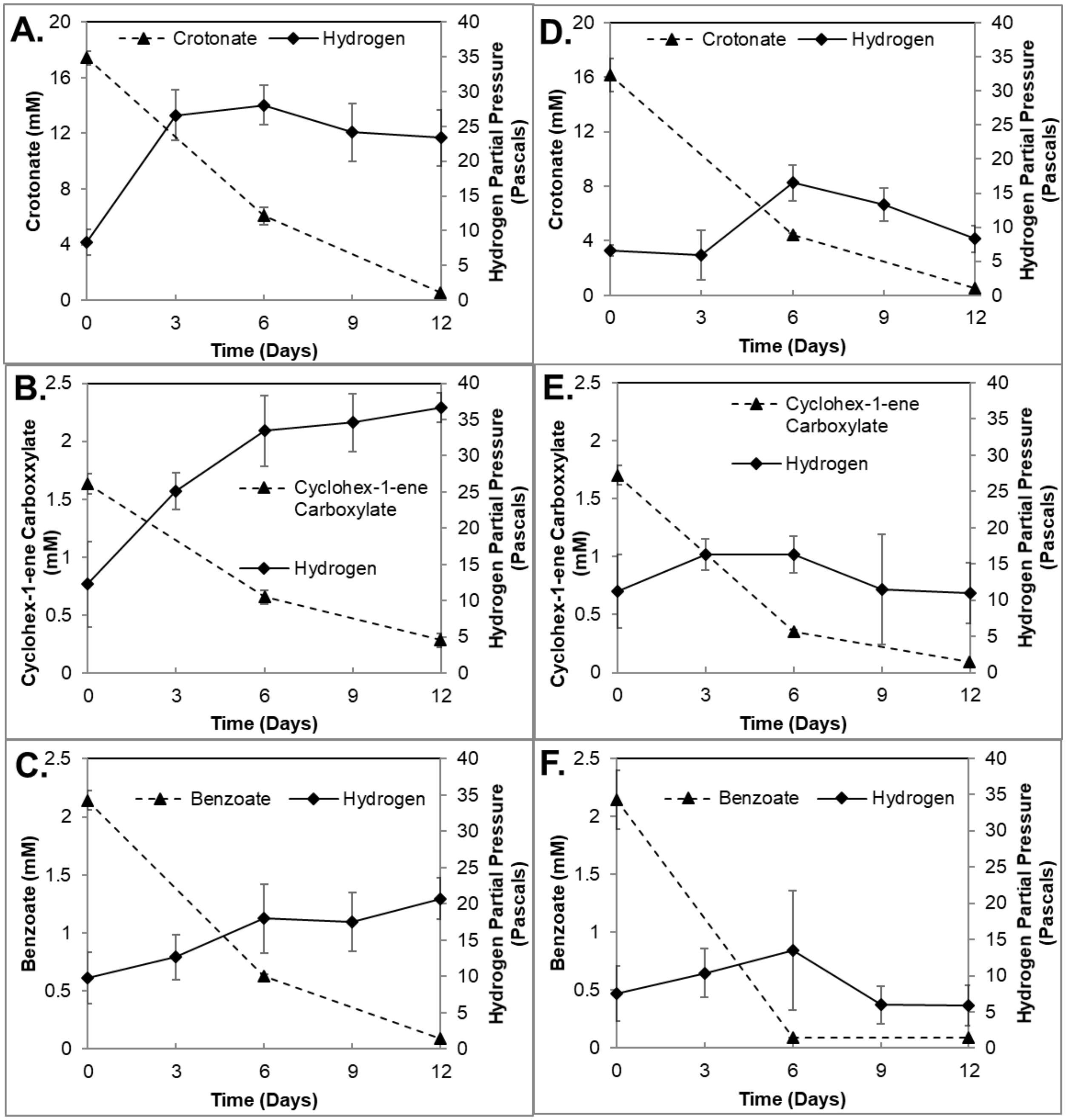

Figure S6. Phylogenetic Comparison of BF and non-BF NADH Dependent Beta Subunits (NqoI/NuoF Homlogs). The tree is drawn to scale with branch lengths measured in the number of substitutions per site. A total of 495 positions were used for this analysis trimmed from a total of 1930 positions (positions 907-1402 in in the full alignment) from the full alignment (Supplemental File 1.).

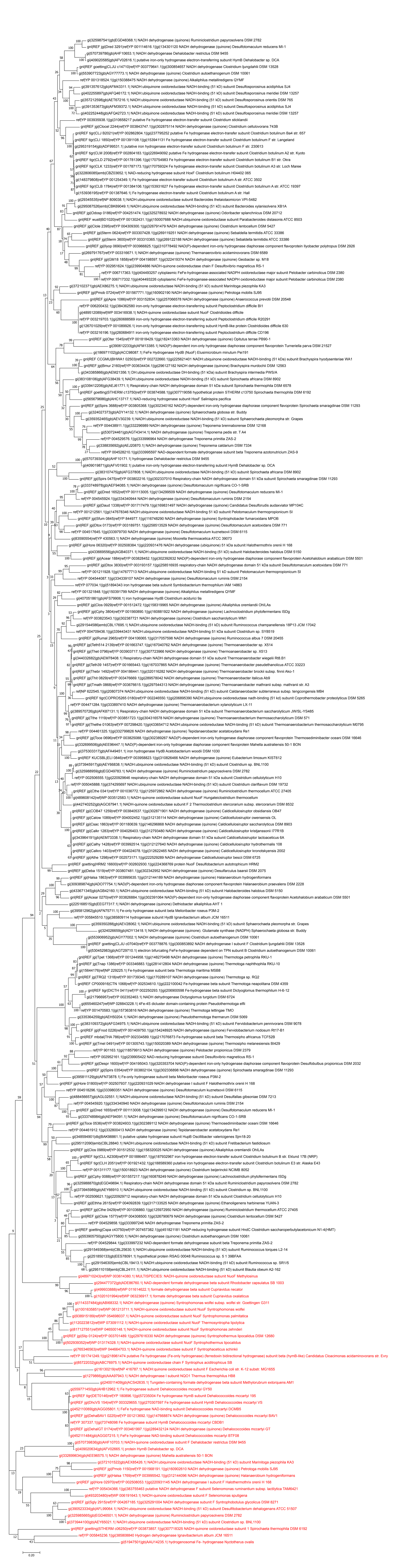

Supplement: Supplementary file 3 [file Data_Sheet_3.pdf]
